# Antihomotypic affinity maturation improves human B cell responses against a repetitive epitope

**DOI:** 10.1126/science.aar5304

**Published:** 2018-06-07

**Authors:** Katharina Imkeller, Stephen W. Scally, Alexandre Bosch, Gemma Pidelaserra Martí, Giulia Costa, Gianna Triller, Rajagopal Murugan, Valerio Renna, Hassan Jumaa, Peter G. Kremsner, B. Kim Lee Sim, Stephen L. Hoffman, Benjamin Mordmüller, Elena A. Levashina, Jean-Philippe Julien, Hedda Wardemann

**Affiliations:** 1 B Cell Immunology, German Cancer Research Institute, Heidelberg, Germany; 2 Faculty of Biosciences, Heidelberg University, Heidelberg, Germany; 3 Program in Molecular Medicine, The Hospital for Sick Children Research Institute, Toronto, ON, Canada; 4 Vector Biology Unit, Max Planck Institute for Infection Biology, Berlin, Germany; 5 Institute of Immunology, University Medical Center Ulm, Ulm, Germany; 6 Institute of Tropical Medicine and German Center for Infection Research, Partner Site Tübingen, University of Tübingen, Tübingen, Germany; 7 Sanaria, Rockville, MD, USA; 8 Departments of Biochemistry and Immunology, University of Toronto, Toronto, ON, Canada

## Abstract

Affinity maturation selects B cells expressing somatically mutated antibody variants with improved antigen-binding properties to protect from invading pathogens.We determined the molecular mechanism underlying the clonal selection and affinity maturation of human B cells expressing protective antibodies against the circumsporozoite protein of the malaria parasite *Plasmodium falciparum* (PfCSP).We show in molecular detail that the repetitive nature of PfCSP facilitates direct homotypic interactions between two PfCSP repeat-bound monoclonal antibodies, thereby improving antigen affinity and B cell activation. These data provide a mechanistic explanation for the strong selection of somatic mutations that mediate homotypic antibody interactions after repeated parasite exposure in humans. Our findings demonstrate a different mode of antigen-mediated affinity maturation to improve antibody responses to PfCSP and presumably other repetitive antigens.

Sporozoites of the human malaria parasite *Plasmodium falciparum* (Pf) express a surface protein, circumsporozoite protein (PfCSP), with an immunodominant central NANP (Asn-Ala-Asn-Pro) repeat region (*1**–**3*). Antibodies against the repeat can mediate protection from *Plasmodium* infection in animal models (*4**–**6*). However, anti-NANP antibody– mediated protection is not readily achieved through vaccination. Thus, the induction of protective PfCSP NANP antibodies is a major goal in pre-erythrocytic vaccine development (*7*). We recently showed that the anti-NANP PfCSPmemory B cell response in Pf-naïve volunteers after immunizationwith live Pf sporozoites under chloroquine prophylaxis (PfSPZ-CVac)matured predominantly through the clonal selection and expansion of potent Pf inhibitory *IGHV3-33*– and *IGKV1-5*– encoded germline antibodies with an 8–amino acid–long immunoglobulin (Ig) light chain k complementarity-determining region 3 (CDR3) (this 8–amino acid CDR3 is hereafter designated KCDR3:8) (*8**,* *9*).

We analyzed five representative germline or low-mutated antibodies with reported affinities for a NANP 5-mer peptide (NANP_5_) between 10^−6^ and 10^−9^ M (Fig. 1A and table S1) (*9*). Antigen binding was abrogated when the original Ig Vκ1-5 light chain was replaced by Vk2-28 or when the native Ig heavy chains were paired with a Vκ1-5 light chain with 9–amino acid–long KCDR3 (Fig. 1B), demonstrating the importance of these specific Ig features in antigen recognition.

**Fig. 1:**
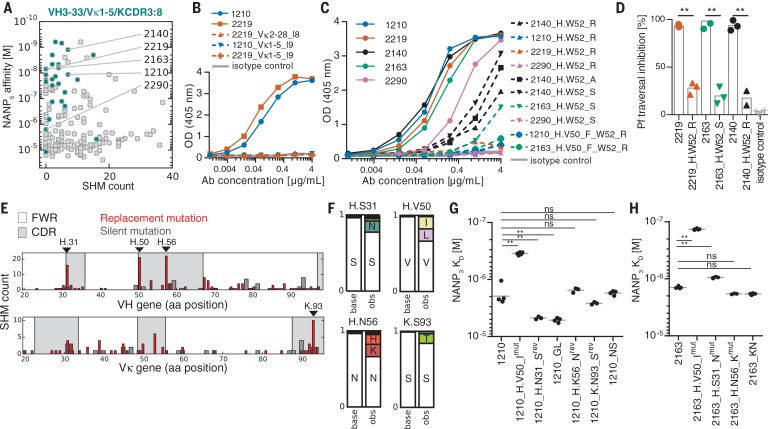
**Affinity maturation of high-affinity human PfCSP NANP antibodies. (A)** Surface plasmon resonance (SPR) affinity and SHM of selected (labeled) V_H_3-33–Vκ1-5–KCDR3:8 (green) and non–V_H_3-33–Vκ1-5–KCDR3:8 (gray) anti-PfCSP antibodies (*9*). (**B** to **D**) Original and mutated antibodies. [(B) and (C)] PfCSP enzymelinked immunosorbent assay reactivity. Data in (A), (B), and (C) are from one experiment representative of at least two independent experiments. OD, optical density; Ab, antibody. Single-letter abbreviations for the amino acid residues are as follows: A, Ala; C, Cys; D, Asp; E, Glu; F, Phe; G, Gly; H, His; I, Ile; K, Lys; L, Leu; M, Met; N, Asn; P, Pro; Q, Gln; R, Arg; S, Ser; T, Thr; V, Val; W, Trp; and Y, Tyr. (D) Pf liver cell traversal inhibition. Bars represent means from two to four independent experiments (symbols represent results from individual experiments). **P = 0.01 (significant) for two-tailed Student’s t test. (**E**) Silent (gray) and replacement (red) SHM (bars) in V_H_3-33–Vκ1-5 antibodies (n = 63). FWR, framework region; aa, amino acid. (**F**) Observed (obs) amino acid usage compared with a baseline (base) model (*22**,* *23*). (**G** and **H**) Independent NANP_3_ SPR affinity measurements (dots) and means (gray lines). **P = 0.01 (significant) for Bonferroni multiple-comparisons test; ns, not significant. KD, equilibrium dissociation constant.

All V_H_3-33–Vκ1-5–KCDR3:8 antibodies were encoded by the IGHV3-33^*^01 allele (*9*). IGHV3- 33^*^01 differs from three otherwise highly similar gene segments (*IGHV3-30, IGHV3-30-3*, and *IGHV3-30-5*) at position 52 of heavy-chain CDR2 (HCDR2), which encodes strictly a tryptophan residue and not serine or arginine (Table 1 and table S2).HCDR2W^52^→S (H.W52_S)andH.W52_R mutants of the selected antibodies, as well as an H.W52_A mutant of antibody 2140 and a double mutant (H.V50_F_W52_R) to mimic the IGHV3- 30*02 and IGHV3-30-5^*^02 alleles, all showed reduced PfCSP repeat reactivity associated with reduced in vitro parasite inhibitory activity (Fig. 1, C and D; single-letter amino acid abbreviations are defined in the legend to Fig. 1).

**Table 1:** HCDR2 residues encoded by different IGHV3-33, IGHV3-30, IGHV3-30-3, and IGHV3-30-5 alleles. Gene and allele data are from www.imgt.org/genedb/.

Gene	Allele(s)	Residue at position			
		50	51	52	52A
***IGHV3-33***	01, 02, 03, 04, 06	V	I	W	Y
***IGHV3-33***	05	V	I	S	Y
***IGHV3-30***	01, 03, 04, 05, 06, 07, 08, 09, 10, 11, 12, 13, 14, 15, 16, 17, 18, 19	V	I	S	Y
***IGHV3-30-3***	01, 02, 03	V	I	S	Y
***IGHV3-30-5***	01	V	I	S	Y

The majority of NANP-reactive V_H_3-33–Vκ1-5– KCDR3:8 B cells belonged to clonally expanded and somatic hypermutation (SHM)–diversified cell clusters with strong selection for replacement mutations in HCDR1 (H.S31) and HCDR2 (H.V50 and H.N56), as well as KCDR3 [KCDR3 S^93^ (K.S93)], likely as a result of affinity maturation (Fig. 1, E and F) (9). The introduction of missing somatic mutations (mut) or reversions (rev) at H.V50 and, to a lesser extent, H.S31 revealed a role in binding to a minimal NANP_3_ peptide (*10**,* *11*), as demonstrated for the germline antibody 2163 and the low-mutated antibody 1210 (Fig. 1, G and H, and table S3). In contrast, exchanges at H.N56 and K.S93, either alone (in antibodies 1210_H.K56_N^rev^, 1210_K. N93_S^rev^, and 2163_H.N56_K^mut^) or in combination (in 1210_NS and 2163_KN), showed no significant effect (Fig. 1, G and H, and table S3). Thus, affinitymaturation to the repeat explained the strong selection for only two of the four characteristic replacementmutations in V_H_3-33– Vκ1-5–KCDR3:8 anti-NANP antibodies.

We next determined the cocrystal structure of the 1210 antigen-binding fragment (Fab) with NANP_5_ (Fig. 2, fig. S1A, and tables S4 to S6). The NANP core epitope contained a type I β turn and an elongated conformation (Fig. 2, A and C, and fig. S1B), similar to NANP bound to a chimeric 2140 Ig heavy chain–1210 Ig k antibody and in line with previous observations (fig. S1C and tables S4 and S7) (*10**–**14*).Main-chain atoms in KCDR3 were optimally positioned to mediate H bonds with the repeat, likely contributing to the strong selection of KCDR3:8 (Fig. 2, B and C, and tables S2, S5, and S10). V_H_3-33 germline residues, notably H.V50 and H.W52 (the residue encoded only by *IGHV3-33* alleles), as well as H.Y52A and H.Y58 in HCDR2, mediated the majority of antigen contacts (table S5 and fig. S2) (*15*). Affinity maturation at H.V50 and H.S31 may be explained by strengthened van der Waals interactions with the repeat (Fig. 2C).

**Fig. 2:**
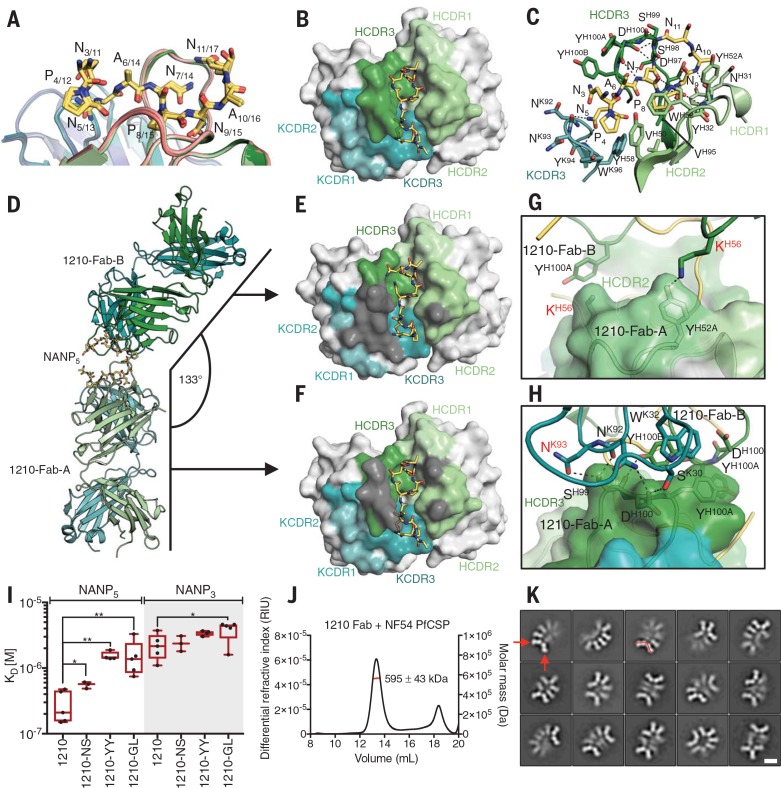
**Affinity maturation drives homotypic repeat binding.** (**A** to **H**) 1210 Fab-NANP_5_ cocrystal structure. (A) Superposition of the four NANP-bound Fabs. (**B**) Surface representation of the antigen-antibody interaction. (**C**) Details of core epitope recognition by 1210. Black dashes indicate H bonds. (**D**) Two 1210 Fabs in complex with NANP_5_. [(**E**) and (**F**)] Surface representations of Fab-B (E) and Fab-A (F). Residues involved in homotypic interactions are dark gray. [(**G**) and (**H**)] Details of homotypic interactions. Affinitymatured residues are labeled in red. (**I**) Mean ± SEM K_D_ determined by isothermal titration calorimetry (ITC). Dots represent independent measurements. One-tailed Mann-Whitney test: *P < 0.05, **P < 0.01. (**J**) Results from size exclusion chromatography coupled with multiangle light scattering (SEC-MALS) for the 1210 Fab–PfCSP complex. The red line indicates mean ± SD molar mass from two measurements. RIU, refractive index units. (**K**) Two-dimensional class averages for the 1210 Fab–PfCSP complex. Red arrows indicate individual Fabs, and red lines indicate the binding angle observed in the crystal structure (D). NF54, Pf strain. Scale bar, 10 nm.

Notably, our crystal structure also revealed that two 1210 Fabs (designated 1210 Fab-A and Fab-B) bound to one NANP_5_ peptide in a headto- head configuration at a 133° angle (Fig. 2D and fig. S3). This binding mode led to six homotypic antibody-antibody H bonds providing 263 Å^2^ of buried surface area (BSA) between the two Fabs and an additional ~120 Å^2^ of BSA between the Fabs and the repeat (Fig. 2, E and F, and tables S5, S6, and S10). Two highly selected mutations, H.N56_K and K.S93_N (Fig. 1, E and F), formed H bonds with H.Y52A and H.S99 in the opposing Fab, thereby stabilizing the head-tohead configuration (Fig. 2, G and H). KCDR3:8 optimally contacted HCDR3 of the opposite 1210 molecule, providing another explanation for the length restriction in KCDR3.

To investigate homotypic interactions, we next measured the Fab affinities for NANP_5_ and NANP_3_ for 1210, 1210_NS (which lacks the selected mutations involved in homotypic binding), a 1210H.D100_Y^mut^ K.N92_Y^mut^ mutant (1210_YY, designed to disrupt head-to-head binding through steric clashes), and a 1210 germline antibody (1210_GL) (Fig. 2I and fig. S4). Compared with 1210, 1210_YY and 1210_NS showed significantly weakened affinity for NANP_5_ but not for NANP_3_, whereas 1210_GL was significantly worse than 1210 at binding both peptides (Fig. 2I and fig. S4) (*16*). These data suggest that only 1210 efficiently recognized the repeat in a high-affinity homotypic head-to-head binding configuration. An analysis of full-length PfCSP with 38 NANP repeats confirmed this hypothesis. Approximately twelve 1210 Fabs bound PfCSP and recognized the NANP repeat in a head-to-head binding configuration similar to the 1210 Fab–NANP_5_ crystal structure (Fig. 2, J and K, and fig. S3D) (*11**,* *17*). Furthermore, 1210_YY IgG, with its restricted ability to engage in homotypic antibody interactions, showed a lower binding avidity to fulllength PfCSP than 1210 (fig. S5). Thus, affinity maturation selects for mutations that improve homotypic antibody interactions, thereby indirectly increasing PfCSP NANP binding.

To better understand the selection of SHM at the cellular level, we measured the degree of B cell activation in response to NANP_5_ of transgenic B cell lines expressing 1210 or variant B cell receptors (BCRs) (Fig. 3, A to D). BCR signaling was delayed in cells expressing 1210_GL compared with that in cells expressing 1210. This effect was even more pronounced in 1210_YY mutant cells. As expected, 1210_H.V50_I^mut^ (1210 with HCDR2 V^50^→I),withhigh repeataffinity,mediated stronger signals than 1210, especially with low antigen concentrations, whereas 1210_NS showed no significant differences (Fig. 3D). Thus, B cell activation is promoted by both direct NANP binding and homotypic antibody interactions. Despite a 2-log difference in NANP_3_ affinities (Fig. 1, G and H) and the varied potential of these antibodies to engage in homotypic interactions, all showed similar capacities to inhibit Pf sporozoites in vitro (Fig. 3E and fig. S6). Likewise, all antibodies conferred similar levels of dose-dependent protection from the development of blood-stage parasites after passive immunization in mice, presumably because of strong avidity effects (Fig. 3F). These data provide a mechanistic explanation for the strong in vivo selection of antihomotypic antibody mutants by affinity maturation, independently of their protective efficacy as soluble antibodies.

**Fig. 3:**
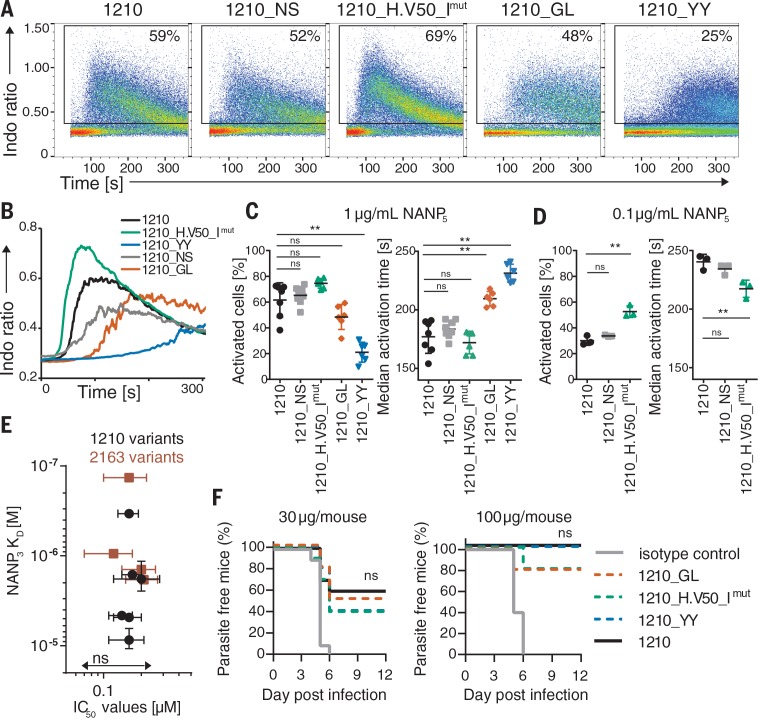
**B cell activation and parasite inhibition.** (**A** to **D**) NANP_5_-induced calcium signaling of 1210 and variants. [(A) and (B)] Reaction kinetics and percentages of activated cells (A) and overlay of median signal intensities (B) with 1 mg/ml NANP5 for one of at least six representative experiments. Indo, calcium indicator. [(C) and (D)] Percentages of activated cells and median activation time after the addition of 1 mg/ml (C) (*n* = 6 or 7 experiments) and 0.1 mg/ml (D) (*n* = 3 experiments) NANP_5_. Symbols indicate results from independent experiments, and lines and error bars indicate means ± SD. **P = 0.01 (significant) for Bonferroni multiple-comparisons test. (**E** and **F**) Parasite inhibition. (E) Mean ± SD median inhibitory concentration (IC_50_) values from at least three independent experiments for 1210 and 2163 antibodies with indicated NANP_3_ affinities.We detected no significant differences between IC_50_ values because of extensively overlapping confidence intervals. (F) Percentages of parasite-free mice after passive immunization with 30 or 100 mg of 1210 or variants 24 hours before subcutaneous injection with *Plasmodium berghei* sporozoites expressing PfCSP (Pb-PfCSP). Data are from one (100 mg) or two (30 mg) independent experiments with five mice per group.We detected no significant differences in survival for 1210 variants (Mantel-Cox test).

V_H_3 antibodies dominate the anti-PfCSP memory response (*9**,* *11**,* *14*). In addition to V_H_3-33– Vκ1-5–KCDR3:8, we observed a cluster of highly mutated, affinity-matured V_H_3-23–Vκ1-5 NANPreactive memory B cell antibodies in our selection (Fig. 4, A andB) (9). Although the NANP_5_-binding mode of a representative V_H_3-23–Vκ1-5 antibody, 1450, was different from that of 1210, it also recognized NANP_5_ in a head-to-head configuration, with HCDR3s in direct juxtaposition and the affinity-matured K.N30 residues forming an H bond between Fab-A and Fab-B (Fig. 4, C to E; fig. S7; and tables S4, S8, and S9). Sequence analysis of the V_H_3-23–Vκ1-5 antibody cluster confirmed enrichment for amino acid exchanges that participate directly in antibody-antigen interactions or antibody-antibody contacts or favor a 1450 paratope conformation optimal for NANP epitope recognition (Fig. 4B).

**Fig. 4:**
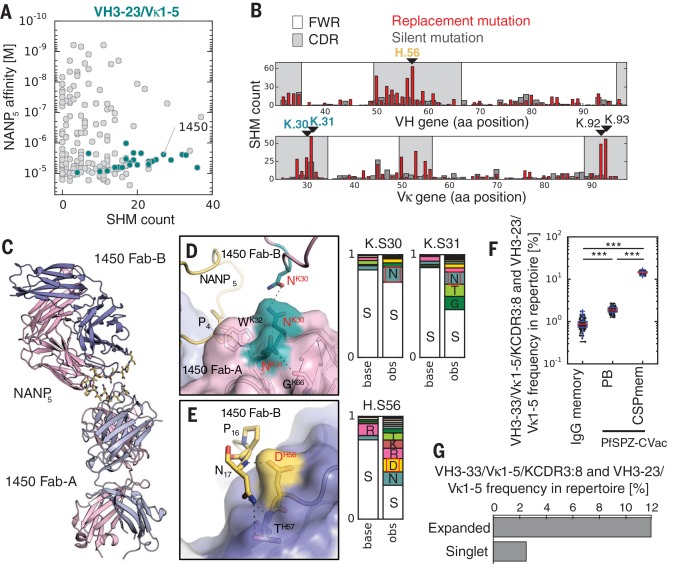
**Antihomotypic affinity maturation in *IGHV3-23*-encoded PfCSP NANP antibodies. (A)** SPR affinity and SHM of 1450 out of all V_H_3-23–Vκ1-5 (green) and non–V_H_3-23–Vκ1-5 (gray) anti-PfCSP antibodies (*9*). (**B**) Silent and replacement SHM (bars) in V_H_3-23–Vκ1-5 antibodies (*n* = 100). (**C** to **E**) Fab 1450–NANP_5_ cocrystal structure. Head-to-head binding mode (C), Fab-Fab (**D**), and Fab-NANP_5_ (E) interactions. Black dashes indicate H bonds. Affinity-matured residues are colored according to the SHM amino acid usage scheme and are labeled in red. Observed amino acid usage is compared with a baseline model (*22**,* *23*). (**F**) V_H_3-33– Vκ1-5–KCDR3:8 or V_H_3-23–Vκ1-5 antibodies in total memory B cells (*18*), CD19^+^CD27^hi^CD38^hi^ plasmablasts (PB), and CD19^+^CD27^+^ PfCSP-reactive memory B cells (CSPmem) (*8**,* *9*). Dots represent subsamples of 1500 sequences. Box plots show the median, SD, maximum, and minimum of the distribution. ***P = 0.001 (significant) for two-tailed Student’s t test. (**G**) Frequency of V_H_3-33–Vκ1-5– KCDR3:8 and V_H_3-23–Vκ1-5 antibodies among clonally expanded versus singlet pooled PB and CSPmem (*9*).

After the immunization of malaria-naïve individuals with PfSPZ-CVac, ~15% of PfCSP-reactive memory B cells showed V_H_3-33–Vκ1-5–KCDR3:8 or V_H_3-23–Vκ1-5 sequence characteristics (Fig. 4F). Furthermore, these cells were strongly enriched in the expanded anti-PfCSP memory B cell pool compared with the nonexpanded population (Fig. 4G). Thus, antihomotypic affinity maturation is observed after repeated Pf sporozoite immunization (*8**,* *9*) in both low-mutated high-affinity V_H_3-33 antibodies and lower-affinity antibodies utilizing other gene combinations. This phenomenon also likely takes place in B cell responses elicited by RTS,Smalaria vaccination (fig. S8) (*11*).

Thus, antihomotypic affinity maturation, in addition to traditional antibody-antigen affinity maturation, promotes the strong clonal expansion and competitive selection of PfCSP-reactive B cells in humans. Even in the absence of affinity maturation, V_H_3-33–Vκ1-5–KCDR3:8 antibodies are moderate to strong NANP binders and potent Pf inhibitors. This critically depends on H.W52 in HCDR2. Because *IGHV3-33* is located in a region of structural polymorphism of the *IGH* locus, haplotype frequencies, especially in areas where Pf is endemic, may determine the efficient induction of protective humoral anti- PfCSP repeat responses upon vaccination (19). Indeed, one donor in our study was *IGHV3-33* negative (fig. S9). We propose that antihomotypic affinity maturation may be a generalizable property of B cell responses if a repetitive antigen (malarial or other) brings two antibodies into close proximity to optimize binding and promote clustering of surface Igmolecules through homotypic interactions (*20**,* *21*).

## Supplementary Material

Antihomotypic affinity maturation improves human B cell responses against a repetitive epitope

## SUPPLEMENTARY MATERIALS

www.sciencemag.org/content/360/6395/1358/suppl/DC1

Tables S1 to S10

References (*24–35*)
