# Supplementary material for: Antihomotypic affinity maturation improves human B cell responses against a repetitive epitope
Source: Science. 2018 Jun 7;360(6395):1358–62. doi: 10.1126/science.aar5304 (PMC6420115; doi:10.1126/science.aar5304)
Supplement: Antihomotypic affinity maturation improves human B cell responses against a repetitive epitope [file Science-360-1358-s1.pdf]

www.sciencemag.org/cgi/content/full/science.aar5304/DC1

Supplementary Materials for

Antihomotypic affinity maturation improves human B cell responses against a repetitive epitope

Katharina Imkeller*, Stephen W. Scally*, Alexandre Bosch, Gemma Pidelaserra Martí, Giulia Costa, Gianna Triller, Rajagopal Murugan, Valerio Renna, Hassan Jumaa, Peter G. Kremsner, B. Kim Lee Sim, Stephen L. Hoffman, Benjamin Mordmüller, Elena Levashina, Jean-Philippe Julien†, Hedda Wardemann†

*These authors contributed equally to this work.
†Corresponding author. Email: h.wardemann@dkfz.de (H.W.); jean-philippe.julien@sickkids.ca (J.-P.J.)

Published 7 June 2018 on *Science* First Release
DOI: 10.1126/science.aar5304

This PDF file includes:

Materials and Methods
Figs. S1 to S9
Tables S1 to S10
References

Materials and Methods

Genotyping

The study was approved by the ethics committee of the medical faculty and the university clinics of the University of Tübingen and strictly adhered to Good Clinical Practice and the principles of the Declaration of Helsinki. The clinical trial from which the samples were obtained was registered under https://clinicaltrials.gov/ct2/show/NCT02115516 and number 2013-003900-38 in the EudraCT database and carried out under FDA IND 15862 and with approval of the Paul-Ehrlich-Institute (*8*, *9*). Genomic DNA was extracted from whole blood. *IGHV3* gene family segments were amplified using barcoded primers. Amplicons were pooled and prepared for sequencing using the TruSeq PCR-free library-prep kit (Illumina). Sequencing was performed on a MiSeq sequencer using a 300-300-bp paired-end protocol. Sequencing reads were assembled using PandaSeq (*24*) and assigned to the donors by barcode identification.

Site-directed mutagenesis

Site-directed mutagenesis on the antibody encoding plasmids was performed using the Q5 site-directed mutagenesis kit (Qiagen).

Antibody and Fab production

For IgG production, *IGH* and *IGK* variable regions were cloned into expression vectors upstream of human *IGK* and *IGG1* constant regions, respectively, as previously described (*25*). Recombinant monoclonal antibodies were expressed in HEK293F cells (ThermoFisher Scientific) and antibody concentrations of Protein G Sepharose (GE healthcare)-purified antibodies were determined by ELISA as previously described (*9*, *10*). Fabs were generated by papain digestion of IgG, purified via Protein A chromatography followed by cation-exchange chromatography (MonoS, GE Healthcare) and size-exclusion chromatography (Superdex 200 Increase 10/300 GL, GE Healthcare). For ITC studies, *IGH* and *IGK* variable regions were cloned into pcDNA3.4 TOPO expression vectors immediately upstream of human *IGK* and *CH1* constant regions, respectively. Fab were transiently expressed in HEK293F cells (ThermoFisher Scientific) and purified via KappaSelect affinity chromatography (GE Healthcare) and size-exclusion chromatography (Superdex 200 Increase 10/300 GL, GE Healthcare).

Antigen production

ELISAs were performed against $NANP_5$ (Alpha Diagnostic International), $NANP_3$ (PSL GmbH, Heidelberg) or PfCSP with an N-terminal truncation expressed in *E. coli* as previously described (*10*, *26*). For BLI, SEC-MALS and single particle negative-stain EM, full length PfCSP (NF54 strain) was cloned into pcDNA3.4-TOPO for transient expression in HEK293F cells. PfCSP was purified via HisTrap Ni/NTA (GE Healthcare) and size-exclusion chromatography (Superdex 200 Increase 10/300 GL, GE Healthcare).

Surface plasmon resonance

Surface plasmon resonance measurements were performed on a BIACORE T200 instrument (GE Healthcare) docked with a series S sensor chip CM5 (GE Healthcare). Ten millimolar HEPES with 150 mM NaCl at pH 7.4 was used as a running buffer as described (*9*). Anti-human IgG antibodies were immobilized on the chip using an amine-coupling based human antibody capture kit. Equal concentrations of sample antibody and isotype control were captured in the sample and the reference flow cells, respectively. Running buffer was injected for 20 min at a rate of 10 µL/min in order to stabilize the flow cells. $NANP_3$ at 0.015, 0.09, 0.55, 3.3, and 20 µM in running buffer was injected at a rate of 30 µL/min. The flow cells were regenerated with 3 M $MgCl_2$. The data were fit by steady-state kinetic analysis using the BIACORE T200 softwareV2.0.

Crystallization and structure determination

Purified 1210 and chimeric H.2140/K.1210 Fabs were concentrated to 12 mg/mL and diluted to 10 mg/mL with $NANP_5$ (10 mg/mL) and $NANP_3$ (10 mg/mL), respectively, in a 1:5 molar ratio prior to crystallization trials. Purified 1450 Fab and $NANP_5$ were mixed in a 3:1 molar ratio and excess 1450 Fab was purified away via size-exclusion chromatography (Superdex 200 Increase 10/300 GL, GE Healthcare). Purified 1450-$NANP_5$ was then concentrated to 6 mg/mL prior to crystallization trials. 1210-$NANP_5$ co-crystals grew in 20% (w/v) PEG 3350 and 0.2 M sodium citrate and were cryoprotected in 15% (w/v) ethylene glycol. Co-crystals of the chimeric H.2140/K.1210 Fab in complex with $NANP_3$ grew in 20% (w/v) PEG 4000, 0.6 M sodium chloride, and 0.1 M MES pH 6.5 and were cryoprotected in 15% (w/v) glycerol. 1450-$NANP_5$ co-crystals grew in 22.5% (w/v) PEG 3350 and 0.2 M di-ammonium hydrogen citrate and were cryoprotected in 15% (w/v) ethylene glycol. Data were collected at the 08ID-1 beamline at the Canadian Light Source (CLS) or at the 23-ID beamline at the Advanced Photon Source (APS), processed and scaled using XDS (*27*). The structures were determined by molecular replacement using Phaser (*28*). Refinement of the structures was carried out using phenix.refine (*29*) and iterations of refinement using Coot (*30*). Software were accessed through SBGrid (*31*).

Isothermal titration calorimetry

Calorimetric titration experiments were performed with an Auto-iTC200 instrument (Malvern) at 25°C. Proteins were dialyzed against 20 mM Tris pH 8.0 and 150 mM sodium chloride overnight at 4°C. $NANP_5$ and $NANP_3$ peptides were diluted in dialysis buffer to 2-3 µM and added to the calorimetric cell, which was titrated with 1210, 1210_GL, 1210 H.D100Y^{mut}_K.N92Y^{mut} (1210_YY), and 1210_H.K56_N^{rev}_K.N93_S^{rev} (1210_NS) Fabs (100 µM) in 15 successive injections of 2.5 µl. Experiments were performed at least three times and the mean and standard error of the mean were reported (Fig. S4). The experimental data were analyzed according to a 1:1 binding model by means of Origin 7.0. Statistical analysis was performed using a one-tailed Mann/Whitney test in Prism.

Biolayer interferometry binding studies

BLI (Octet RED96, ForteBio) experiments were conducted to determine the binding avidity of 1210 and 1210_YY IgG for full length PfCSP. Full-length PfCSP was diluted to 10 μg/mL in kinetics buffer (PBS, pH 7.4, 0.01 % (w/v) BSA, and 0.002% Tween20) and immobilized onto Ni/NTA (NTA) biosensors (FortéBio). Following the establishment of a stable baseline with loaded ligand in kinetics buffer, biosensors were dipped into wells containing twofold dilution series of IgG. Tips were then dipped back into kinetics buffer to monitor the dissociation rate. Kinetics data were analyzed using FortéBio's Data Analysis software 9.0, and curves were fitted to a 1:1 binding model.

Size-exclusion chromatography-multi-angle light scattering (SEC/MALS)

$NANP_5$ peptide was co-complexed with a threefold molar excess of 1210 Fab and loaded on a Superdex 200 Increase 10/300 GL (GE Healthcare), coupled in-line to an AKTA Pure chromatography system (GE Healthcare) with the following calibrated detection systems: (i) MiniDawn Treos MALS detector (Wyatt); (ii) Quasielastic light scattering (QELS) detector (Wyatt); and (iii) Optilab T-reX refractive index (RI) detector (Wyatt). Three hundred thirty micrograms of full-length PfCSP was loaded on a Superdex 200 Increase 10/300 GL (GE Healthcare), coupled in-line with an Agilent Technologies 1260 Infinity II HPLC with the detection systems described above. Full-length PfCSP (5 μM) was co-complexed with a 20-fold molar excess of 1210 Fab (100 μM) and either 100 μL or 400 μL was loaded on a Superose 6 Increase 10/300 GL (GE Healthcare) in-line with an Agilent Technologies 1260 Infinity II HPLC with the detection systems described above. Data processing was performed using the ASTRA software (Wyatt).

Negative-stain transmission electron microscopy

400 mesh Cu grids were coated with colloidon and a thin continuous layer of carbon was evaporated onto the grids. Carbon grids were glow discharged according to standard protocols. A 3-μL drop of co-complexed 1210 Fabs with full-length PfCSP was applied to a glow-discharged carbon grid. After 20 s, the grid was blotted and 3 μL of 1 % (w/v) uranyl formate solution was added three times for two lots of 5 s and a final 18 s, with blots in between. Data were collected on a FEI Tecnai 20 operated at 200 kV. One hundred twenty images were collected with a defocus value between 1 and 3 μm. Initially, a total of 1080 particle images were manually selected with Relion 2.0 (*32*) and 2D classification of particle images was performed with 10 classes allowed. Subsequently, the best six 2D classes comprising 947 particle images were used for autopicking 13,146 particle images from 120 micrographs and 2D classification was performed with 50 classes allowed.

Retroviral transduction of TKO-EST cells

Triple Rag2, λ5, and SLP-65 TKO-EST deficient murine pre-B cells, which lack endogenous BCR expression, were reconstituted with Ig heavy and light chain genes via retroviral transduction (*33*)**.** For the generation of viral particles, constructs encoding complete *IGHM* and *IGκ* variable regions were cloned into the pMIZCC and pMIZYN vector backbones (*34*). 1.8 x 10^5 Phoenix-Eco viral packaging cells per well were seeded into six-well culture plates in complete Iscove's modified Dulbecco's medium (IMDM, including 5% FCS, 2 mM L-glutamine, 0.5 mL β-mercaptoethanol, and penicillin/streptomycin). Twenty-four hours later, cells were transfected with 0.5 µg of heavy-chain and 0.5 µg of light-chain plasmid, in 100 µl of pure IMDM using 3 µl of GeneJuice reagent and incubated for 48 h at 37°C and 8% CO_2. Supernatants were harvested and **v**iral particles were purified using a 0.45-µm filter. 1 µl/mL of polybrene was added to the viral particle suspension. In parallel, 2 x 10^5 TKO-EST cells were transferred into a 1.5-mL tube and centrifuged (366 x *g*, 4°C, 5 min). The supernatant was discarded and the cell pellet was resuspended in 800 µl of the viral particle suspension. TKO-EST cells were spin-transduced at 366 x *g* and 37°C. After 3 h, the medium was replaced with fresh complete IMDM supplemented with IL-7 and the cells were seeded into six-well plates.

Ca^{2+} flux measurement

Ca^{2+} flux was measured as described in (*33*). After viral transduction, 1 × 10^6 TKO-EST cells were loaded for 45 min at 37°C with the calcium-sensitive dye Indo-1 AM (Molecular Probes). The Indo-1 staining solution was prepared by mixing 25 µl of the Indo-1 stock solution (prepared by diluting 50 µg of Indo-1 in 25 µl of DMSO) with 25 µl pluronic acid F-127 and 113 µl of FCS and incubated (5 min, darkness, RT). Indo-loaded cells were washed in 5 mL of 1% FCS IMDM, resuspended in 500 µl of 1% FCS IMDM and transferred into FACS tubes. Each sample was pre-warmed individually for 10 min at 37°C on a hotplate before measurement. After recording the Ca^{2+} flux baseline on a LSR cytometer for 30 s, 5 µl of the antigen solution containing 4-hydroxytamoxifen (4-OHT, final concentration: 2 µM) was added and the Ca^{2+} flux in response to antigen was recorded for 6 min. Surface Ig expression in the different cell lines was comparable when measured in FACS by binding of anti-IgM and anti-IgK fluorescently labelled antibodies. Comparable functionality of all cell lines was confirmed upon stimulation with 4-OHT and the α-Igκ antibody (1 µg/mL).

Pf traversal assay

Pf traversal assays were performed in 96-well-plate format as described (*9*, *10*). In brief, 75,000 Pf sporozoites obtained from female *Anopheles coluzzii* mosquito salivary glands were pre-incubated with different concentrations of monoclonal antibodies for 30 min before incubation with HC-04 human hepatocyte cells in the presence of 0.5 mg/mL dextran/rhodamine (Molecular Probes). Untreated sporozoites and dextran/rhodamine alone were used as positive control and to determine the experimental background signal, respectively. Upon fixation with 1%

paraformaldehyde (PFA), the percentage of dextran-positive (i.e., traversed cells) was measured using an LSR II flow cytometer. The background signal was subtracted from all measurements. Traversal inhibition was determined based on the traversal rate observed for untreated sporozoites. Data for each antibody was pooled from at least three independent experiments and the titration curve fitted using a three-parametric Hill function.

Mouse immunizations and infections

All animal experiments were approved by LAGeSo, Berlin, Germany (H0027/12). Immunizations and infections were performed as previously described (*9*, *10*). In brief, 8-week-old C57BL/6 female mice (5 per group) were passively immunized intraperitoneally with 100 μg or 30 μg of monoclonal human anti-PfCSP antibody or an isotype control (mGO53 (*35*)) in 100 μl of PBS. Twenty-four hours post passive immunization, mice were infected with 5,000 PfCSP transgenic *Plasmodium berghei* (*Pb*-*Pf*CSP) (*10*) sporozoites by subcutaneous injection at the tail base. Giemsa-stained blood smears were analyzed daily from day 3 to day 12 post-infection. At least 100 microscopic fields were counted to declare parasite positivity.

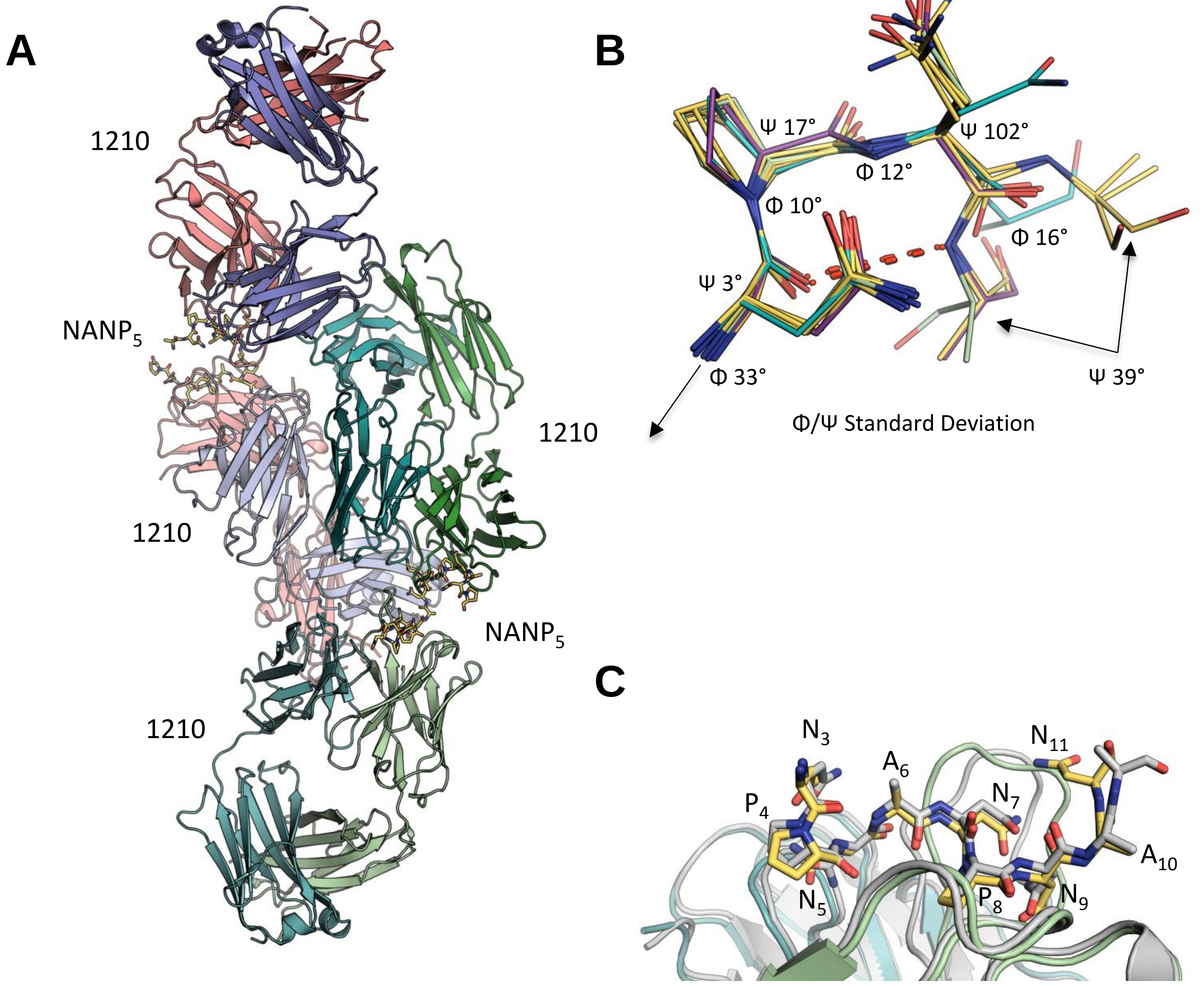

Fig. S1: NANP$_5$ repeat binding by antibody 1210.
A, The four 1210 Fabs bound to 2 NANP$_5$ peptides in the asymmetric unit of the 1210-NANP$_5$ crystal structure. **B**, Superposition of the NPNA cadence of 580 (teal; *(10)*), 663 (green; *(10)*), 1210 (yellow) and the unliganded peptide (purple; (*12)*) structures. The standard deviation in the Phi and Psi angles is shown. **C**, Superposition of 1210-NANP$_5$ with the H.2140 / L.1210 chimeric Fab in complex with a NANP$_3$ peptide. The 1210 bound NANP$_5$ peptide is colored yellow, and the chimeric Fab and NANP$_3$-bound peptide are colored gray.

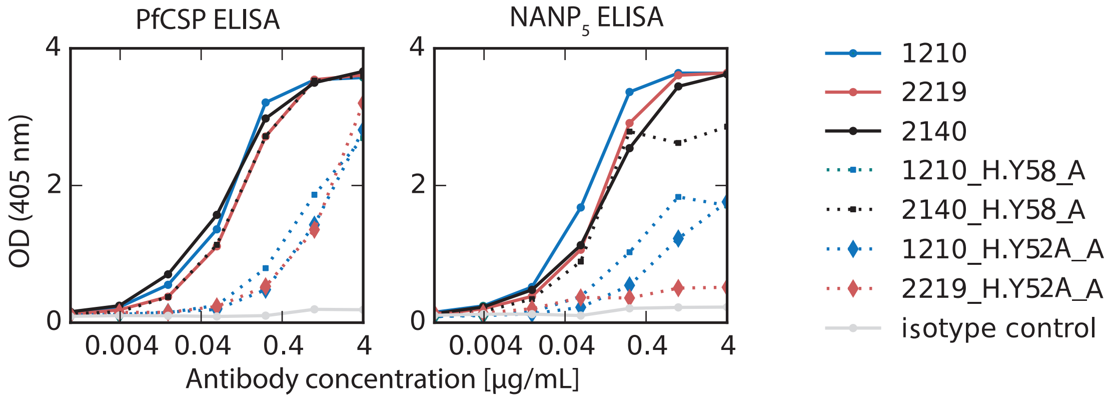

Fig. S2: Effect of alanine exchange of residues H.Y52A and H.Y58 on antigen binding. PfCSP and $NANP_5$ ELISA reactivity of antibodies 1210, 2140, 2219 and respective mutants with alanine exchanges at positions H.Y52A and H.Y58. One out of three representative experiments is shown.

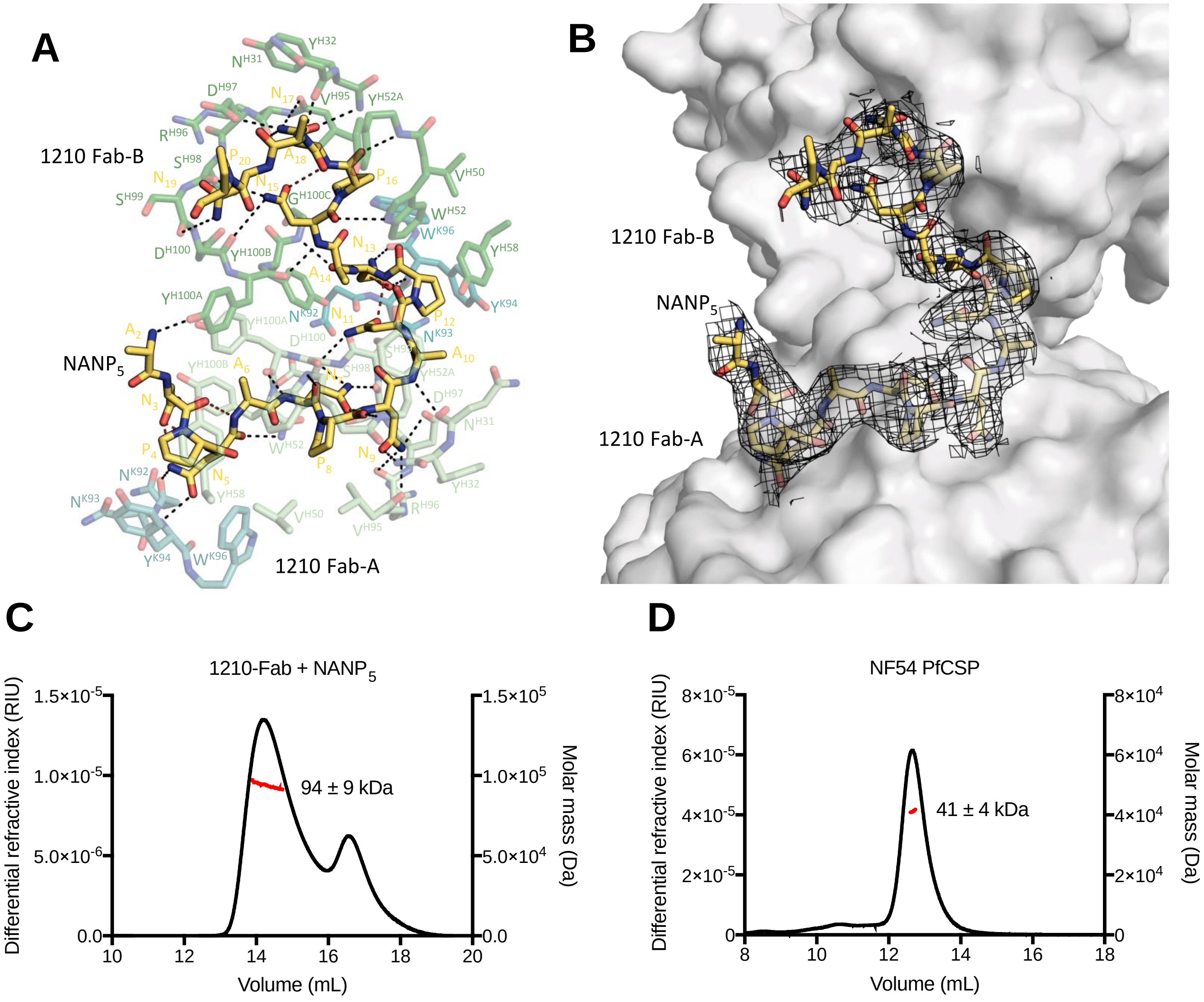

Fig. S3: 1210-NANP$_5$ crystal structure.

A, Detailed interactions of 1210 with NANP$_5$. Intermolecular H-bonds are colored as black dashes and intramolecular H-bonds are colored red. **B**, Unbiased electron density omit map (black mesh) contoured to 1.0 σ for the NANP$_5$ peptide bound to two 1210 Fabs.

C, Elution profile of 1210-NANP$_5$ examined by SEC/MALS. The horizontal red line corresponds to the calculated molar mass for two 1210 Fabs bound to NANP$_5$. **D**, Elution profile of full-length PfCSP examined by SEC/MALS. The horizontal red line corresponds to the calculated molar mass of the eluting antigen.

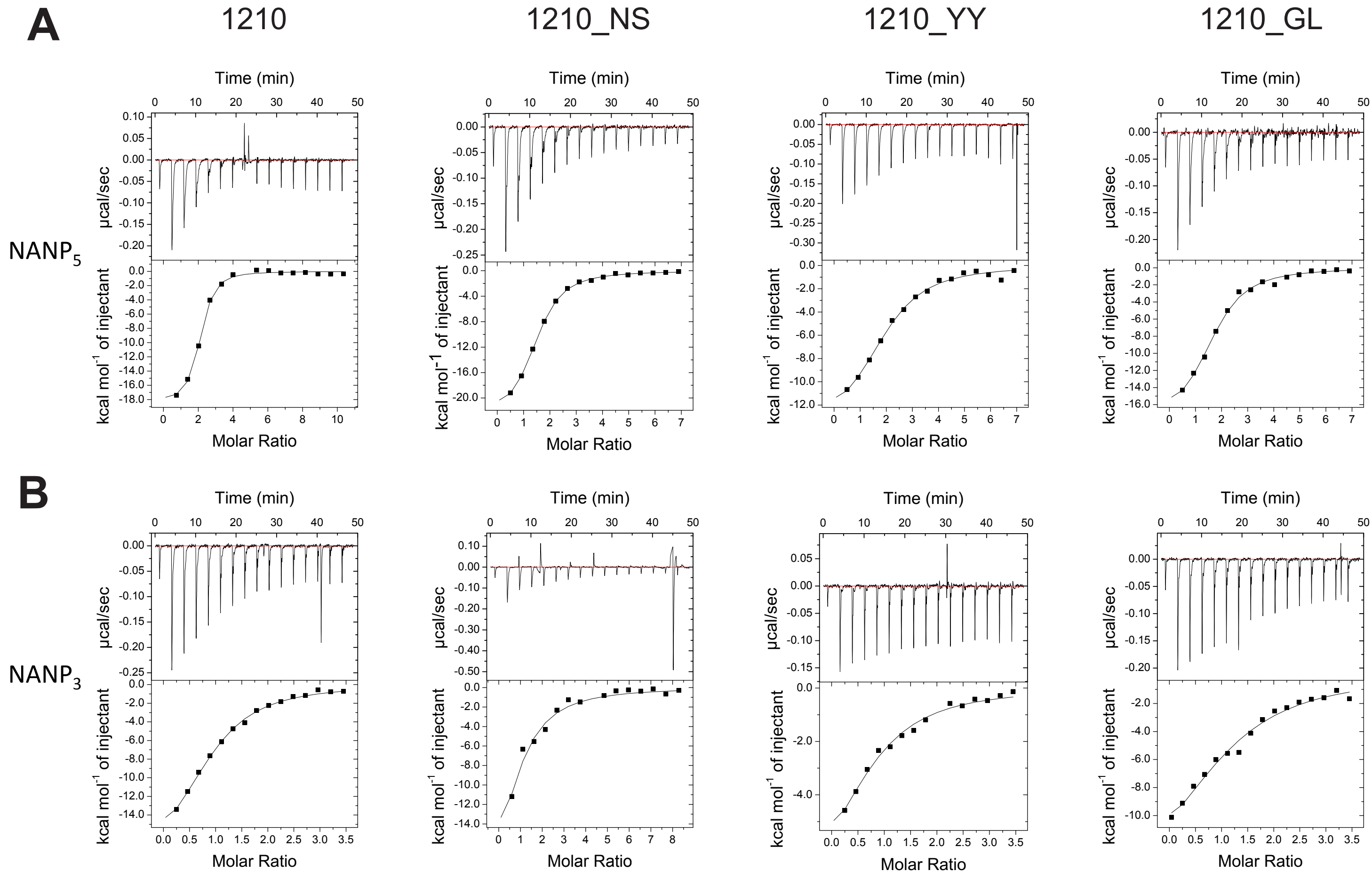

C

Fab	Peptide	Stoichiometry (N)	K_D (nM)	ΔH (kcal/mol)
1210	NANP$_5$	1.8 ± 0.09	286 ± 70	-20 ± 1
1210_NS	NANP$_5$	1.7 ± 0.24	556 ± 36	-22 ± 1
1210_YY	NANP$_5$	2.1 ± 0.12	1538 ± 118	-14 ± 1
1210_GL	NANP$_5$	1.9 ± 0.12	1576 ± 456	-23 ± 5
1210	NANP$_3$	0.73 ± 0.09	2260 ± 446	-21 ± 3
1210_NS	NANP$_3$	0.94 ± 0.05	2420 ± 370	-27 ± 5
1210_YY	NANP$_3$	0.88 ± 0.11	3405 ± 141	-7 ± 0.7
1210_GL	NANP$_3$	0.92 ± 0.05	3858 ± 573	-12 ± 2

Fig. S4: Isothermal titration calorimetry of 1210 binding to NANP repeat peptides.
A, B, Representative raw ITC data (top panel) and fitted binding curves (bottom panel) are shown for 1210, 1210_NS, 1210_YY and 1210_GL binding to NANP$_5$ (A) and NANP$_3$ (B). C, Summary of measured binding thermodynamic values for these interactions observed in (A) and (B). Mean ± SEM for at least three independent experiments is reported.

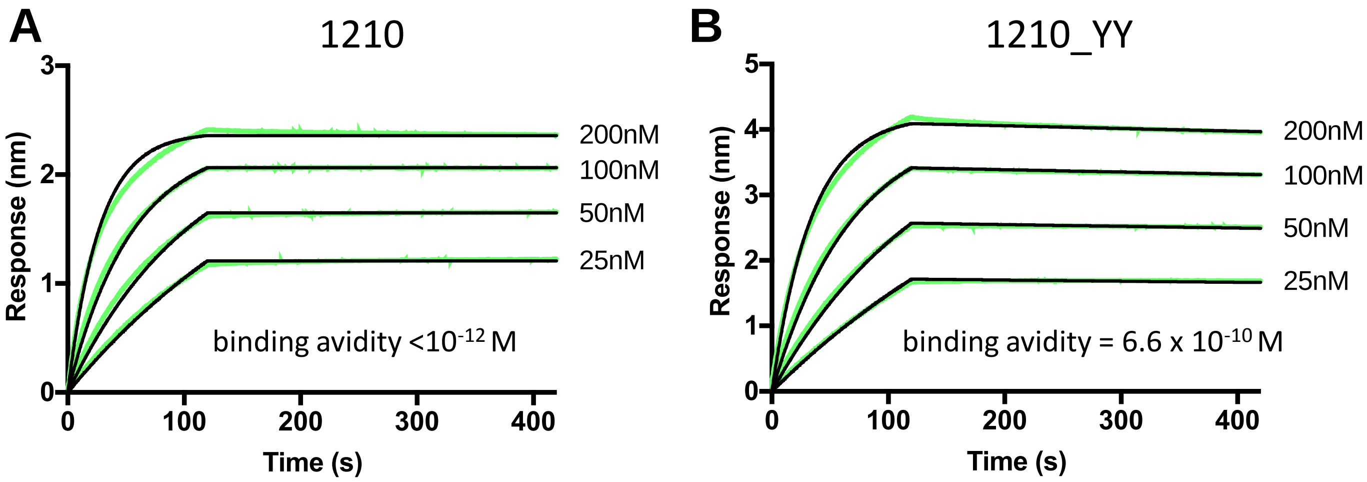

Fig. S5: Binding avidity of 1210 and 1210_YY to PfCSP.
Representative biolayer interferometry sensorgrams (green), 1:1 model best fits (black) and calculated binding avidity for **(A)** 1210 IgG and **(B)** 1210_YY IgG binding to full length PfCSP.

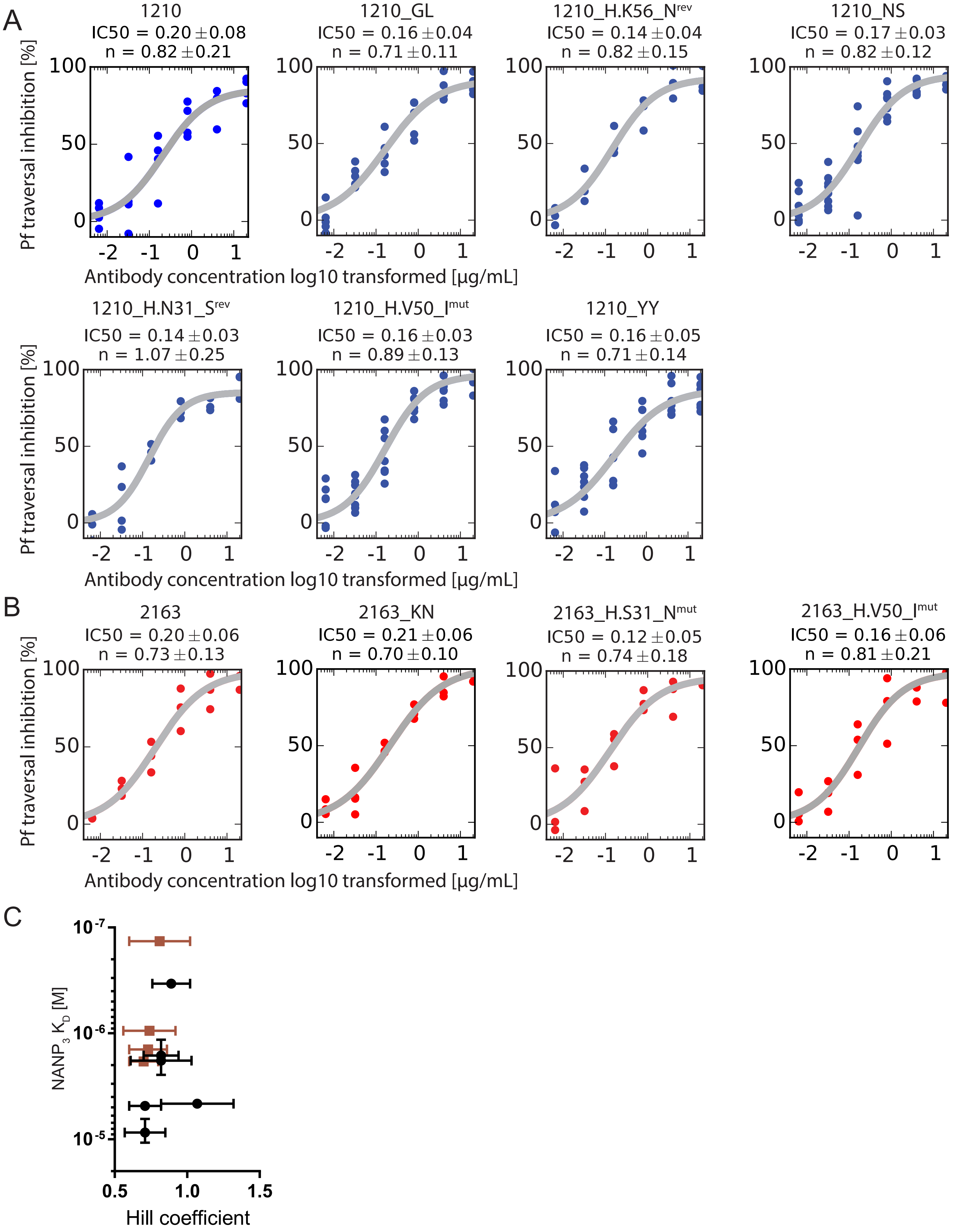

Fig. S6: Antibody mediated inhibition of Pf hepatocyte traversal.
A, **B**, Pf hepatocyte traversal inhibition for 1210 (A), 2163 (B), as well as the indicated variants. The IC_{50} values (in μg/mL) and Hill coefficient (n) values and their standard deviations are indicated above each plot. **C**, $NANP_3$ affinities and Hill coefficient for 1210 (black) and 2163 (brown) as well as the respective variants as shown in (A and B). Error bars indicate standard deviation.

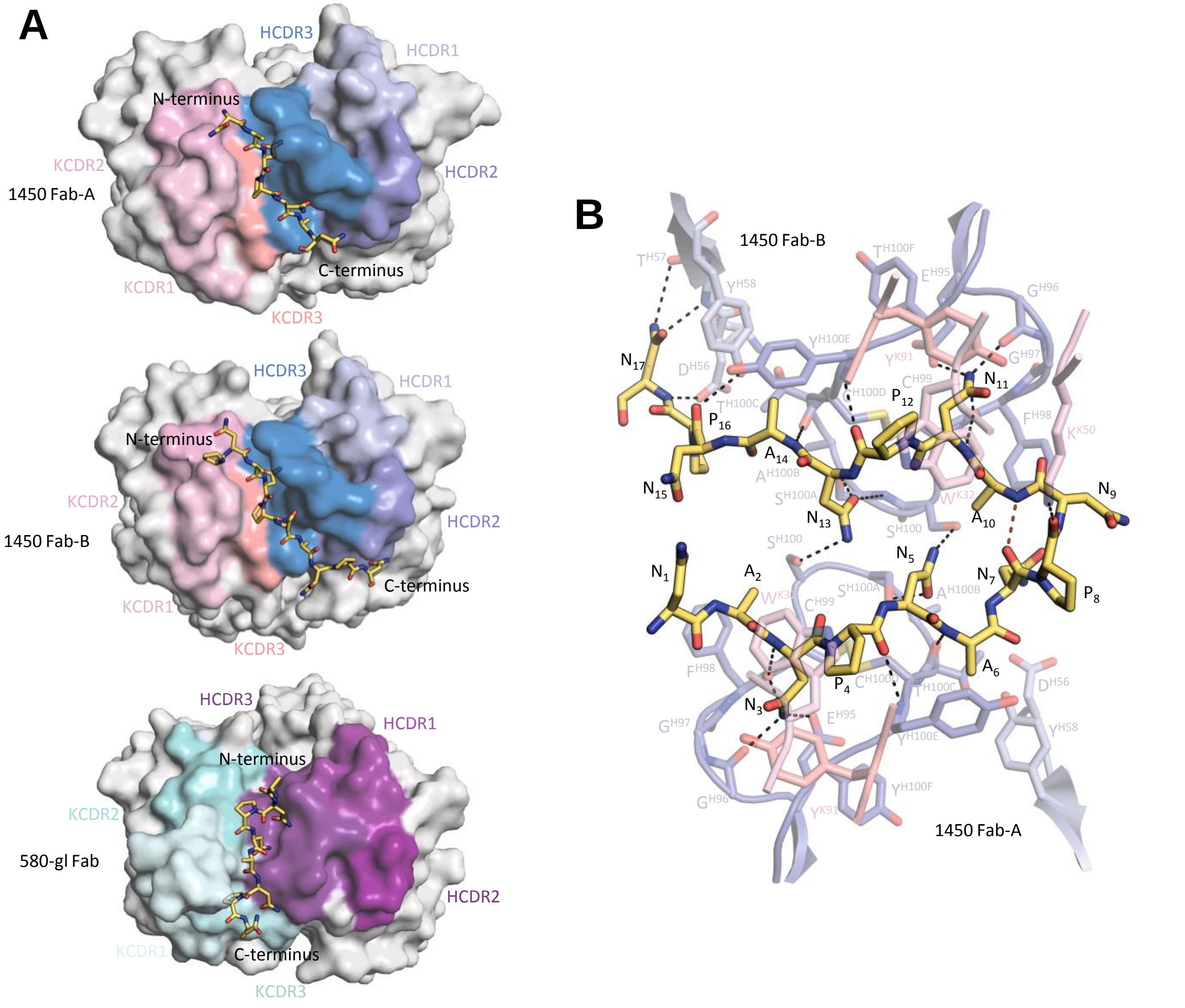

Fig. S7: NANP$_5$ repeat binding by antibodies 1450 and 580-gl.
A, Surface representation of the 1450 and 580-gl (PDB 6AZM, (*10*)) paratopes bound to NANP$_5$. **B,** Detailed interactions of 1450 with NANP$_5$. Intermolecular H-bonds are colored as black dashes and intramolecular H-bonds are colored red.

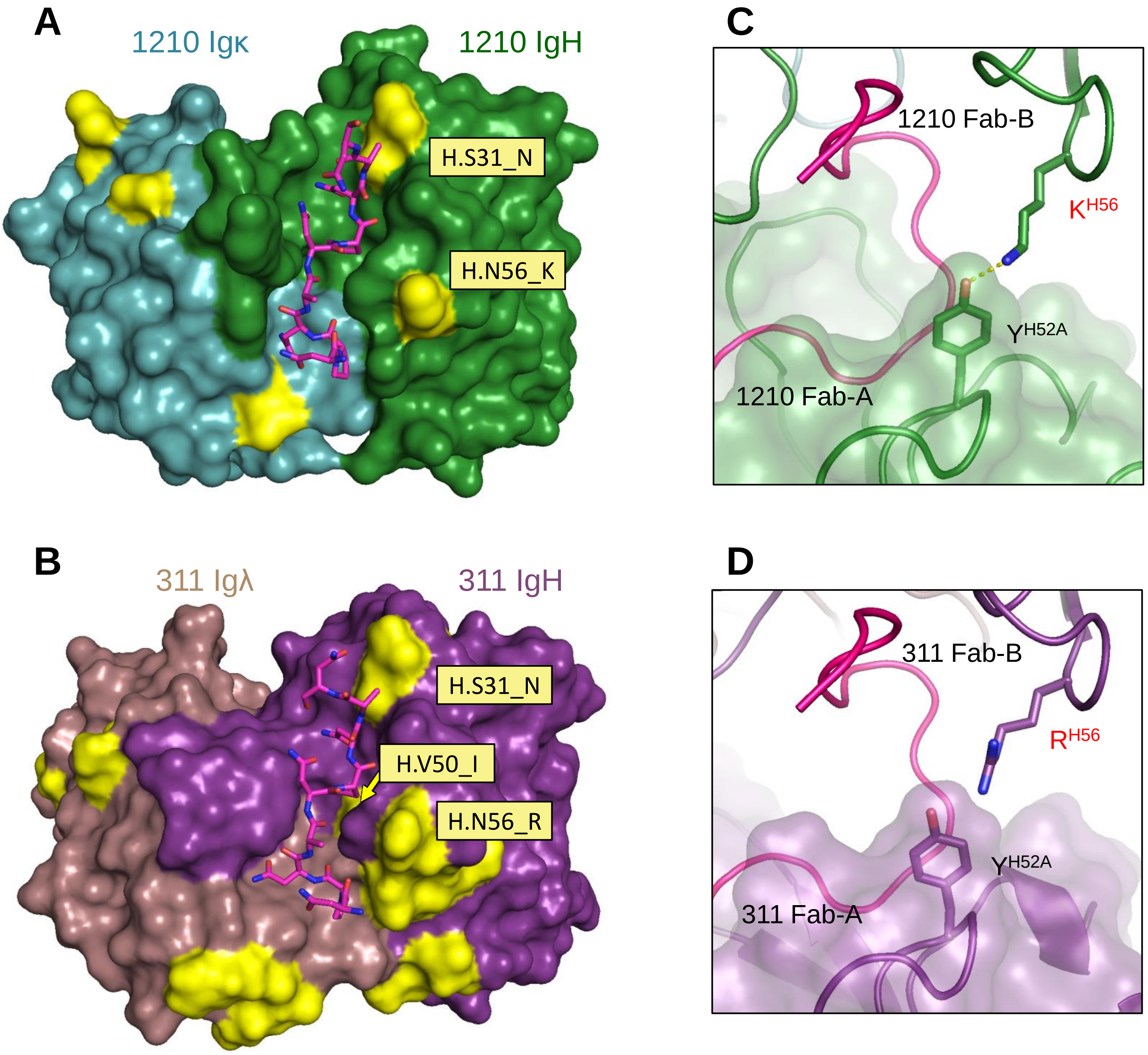

Fig. S8: Structure comparison of 1210 and the RTS,S vaccine-induced NANP antibody 311 (encoded by IGHV3-33 and IGLV1-40).

Similar antigen-binding conformations are observed for recognition of the minimal NPNANPNANA repeat epitope. Analogous to the anti-homotypic mutation H.N56_K in 1210, 311 possesses H.N56_R, suggesting that it may also have undergone anti-homotypic affinity maturation. **A**, 1210 Igκ chain is shown in teal, 1210 IgH chain is shown in green. **B**, 311 Igλ chain is shown in brown, 311 IgH chain is shown in purple. NANP repeat antigens are shown in pink. Mutated residues are colored in yellow. AA-exchanges at positions H.31, H.50 and H.56 are highlighted. **C**, **D**, Detailed representation of homotypic HCDR2 interactions between 1210 (**C**) and 311 (**D**) Fabs binding neighboring repeat epitopes. For D the structure of the 311-NANP complex was duplicated and structurally aligned to both Fab-A and Fab-B of the 1210_NANP5 complex. Affinity matured residues H.K56 (**C**, 1210 Fab) and H.R56 (**D**, 311 Fab, (*11*)) are labeled in red.

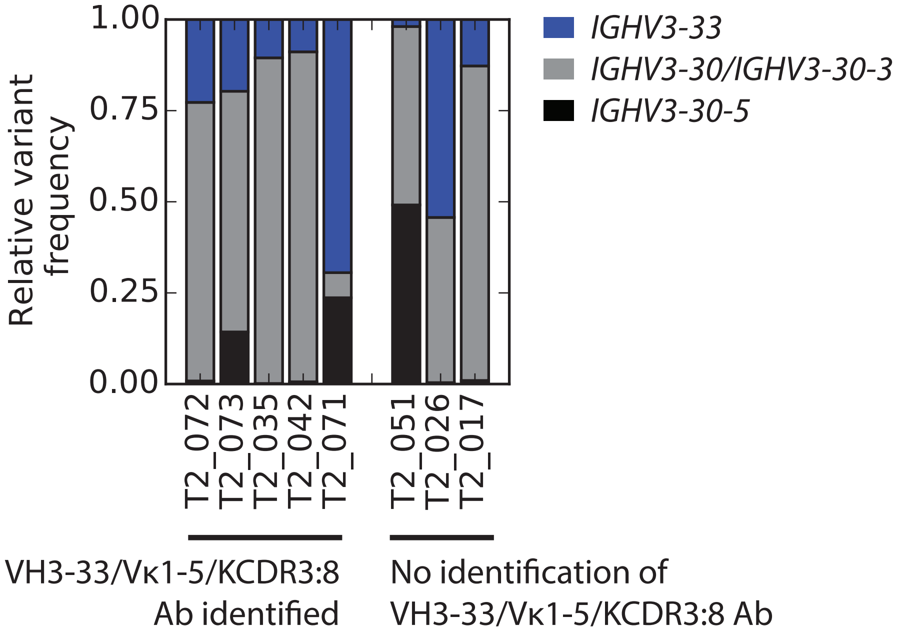

Fig. S9: *IGHV3-33, IGHV3-30/IGHV3-30-3, IGHV3-30-5* gene frequency.
Frequency of *IGHV3-33, IGHV3-30/IGHV3-30-3, IGHV3-30-5* germline gene segments (*8*,*9*) as determined by genomic sequencing of peripheral blood mononuclear cells. Sequences were assigned to the respective germline gene based on their CDR2 sequence as shown in Table 1.

Table S1: VH3-33/VK1-5/K:8 antibody genes features

mAb	*IGHV*	*IGHJ*	H CDR3	*IGKV*	*IGKJ*	KCDR3	Isotype	Replacement SHM	
								H	K
2290	*IGHV3-33*	*IGHJ3*	ARVQDSEDYGGNSGAFDI	*IGKV1-5*	*IGKJ4*	QQYNSYFT	*IGHM*	0	0
1210	*IGHV3-33*	*IGHJ3*	ARVRDSSDYYGDAFDI	*IGKV1-5*	*IGKJ1*	QQYNNYWT	*IGHM*	2	3
2163	*IGHV3-33*	*IGHJ4*	ARVQTTTGGGSCCPFDY	*IGKV1-5*	*IGKJ1*	QQYNSYWT	*IGHM*	0	0
2219	*IGHV3-33*	*IGHJ3*	ARVQDSEDYGGNSGVFDI	*IGKV1-5*	*IGKJ4*	QQYNSYFT	*IGHM*	4	1
2140	*IGHV3-33*	*IGHJ5*	AKVGEGQVGDSSGYYDH	*IGKV1-5*	*IGKJ5*	QQYKSFWT	*IGHG1*	4	1

Table S2: Amino acid sequence of VH3-33, VH3-30, VH3-30-3, VH3-30-5

```
CLUSTAL O(1.2.4) multiple sequence alignment


AB019439|IGHV3-33*01|       QVQLVESGGGVVQPGRSLRLSCAASGFTFSSYGMHWVRQAPGKGLEWVAVIWYDGSNKYY    60
M99665|IGHV3-33*02|         QVQLVESGGGVVQPGRSLRLSCAASGFTFSSYGMHWVRQAPGKGLEWVAVIWYDGSNKYY    60
M77305|IGHV3-33*03|         QVQLVESGGGVVQPGRSLRLSCAASGFTFSSYGMHWVRQAPGKGLEWVAVIWYDGSNKYY    60
M77335|IGHV3-33*04|         QVQLVESGGGVVQPGRSLRLSCAASGFTFSSYGMHWVRQAPGKGLEWVAVIWYDGSNKYY    60
M77334|IGHV3-33*05|         QVQLVESGGGVVQPGRSLRLSCAASGFTFSSYGMHWVRQAPGKGLEWVAVISYDGSNKYY    60
HM855436|IGHV3-33*06|       QVQLVESGGGVVQPGRSLRLSCAASGFTFSSYGMHWVRQAPGKGLEWVAVIWYDGSNKYY    60
M83134|IGHV3-30*01|         QVQLVESGGGVVQPGRSLRLSCAASGFTFSSYAMHWVRQAPGKGLEWVAVISYDGSNKYY    60
L26401|IGHV3-30*02|         QVQLVESGGGVVQPGGSLRLSCAASGFTFSSYGMHWVRQAPGKGLEWVAFIRYDGSNKYY    60
M99663|IGHV3-30*03|         QVQLVESGGGVVQPGRSLRLSCAASGFTFSSYGMHWVRQAPGKGLEWVAVISYDGSNKYY    60
L06615|IGHV3-30*04|         QVQLVESGGGVVQPGRSLRLSCAASGFTFSSYAMHWVRQAPGKGLEWVAVISYDGSNKYY    60
M77323|IGHV3-30*05|         QVQLVESGGGVVQPGRSLRLSCAASGFTFSSYGMHWVRQAPGKGLEWVAVISYDGSNKYY    60
L06617|IGHV3-30*06|         QVQLVESGGGVVQPGRSLRLSCAASGFTFSSYGMHWVRQAPGKGLEWVAVISYDGSNKYY    60
L06614|IGHV3-30*07|         QVQLVESGGGVVQPGRSLRLSCAASGFTFSSYAMHWVRQAPGKGLEWVAVISYDGSNKYY    60
M62737|IGHV3-30*08|         QVQLVDSGGGVVQPGRSLRLSCAASAFTFSSYAMHWVRQAPGKGLEWVAVISYDGSNKYY    60
M77300|IGHV3-30*09|         QVQLVESGGGVVQPGRSLRLSCAASGFTFSSYAMHWVRQAPGKGLEWVAVISYDGSNKYY    60
M77326|IGHV3-30*10|         QVQLVESGGGVVQPGRSLRLSCAASGFTFSSYAMHWVRQAPGKGLEWVAVISYDGSNKYY    60
M77331|IGHV3-30*11|         QVQLVESGGGVVQPGRSLRLSCAASGFTFSSYAMHWVRQAPGKGLEWVAVISYDGSNKYY    60
M77338|IGHV3-30*12|         QVQLVESGGGVVQPGRSLRLSCAASGFTFSSYGMHWVRQAPGKGLEWVAVISYDGSNKYY    60
M77339|IGHV3-30*13|         QVQLVESGGGVVQPGRSLRLSCAASGFTFSSYGMHWVRQAPGKGLEWVAVISYDGSNKYY    60
M77324|IGHV3-30*14|         QVQLVESGGGVVQPGRSLRLSCAASGFTFSSYAMHWVRQAPGKGLEWVAVISYDGSNKYY    60
M77327|IGHV3-30*15|         QVQLVESGGGVVQPGRSLRLSCAASGFTFSSYAMHWVRQAPGKGLEWVAVISYDGSNKYY    60
M77328|IGHV3-30*16|         QVQLVESGGGVVQPGRSLRLSCAASGFTFSSYAMHWVRQAPGKGLEWVAVISYDGSNKYY    60
M77329|IGHV3-30*17|         QVQLVESGGGVVQPGRSLRLSCAASGFTFSSYAMHWVRQAPGKGLEWVAVISYDGSNKYY    60
X92214|IGHV3-30*18|         QVQLVESGGGVVQPGRSLRLSCAASGFTFSSYGMHWVRQAPGKGLEWVAVISYDGSNKYY    60
L06616|IGHV3-30*19|         QVQLVESGGGVVQPGRSLRLSCAASGFTFSSYGMHWVRQAPGKGLEWVAVISYDGSNKYY    60
AC244456|IGHV3-30-3*01|     QVQLVESGGGVVQPGRSLRLSCAASGFTFSSYAMHWVRQAPGKGLEWVAVISYDGSNKYY    60
M77302|IGHV3-30-3*02|       QVQLVESGGGVVQPGRSLRLSCAASGFTFSSYAMHWVRQAPGKGLEWVAVISYDGSNKYY    60
KC713945|IGHV3-30-3*03|     QVQLVESGGGVVQPGRSLRLSCAASGFTFSSYAMHWVRQAPGKGLEWVAVISYDGSNKYY    60
AC244456|IGHV3-30-5*01|     QVQLVESGGGVVQPGRSLRLSCAASGFTFSSYGMHWVRQAPGKGLEWVAVISYDGSNKYY    60
AC245243|IGHV3-30-5*02|     QVQLVESGGGVVQPGGSLRLSCAASGFTFSSYGMHWVRQAPGKGLEWVAFIRYDGSNKYY    60
                            *****:********* *********.******.****************.* ********

AB019439|IGHV3-33*01|       ADSVKGRFTISRDNSKNTLYLQMNSLRAEDTAVYYCAR  98
M99665|IGHV3-33*02|         ADSAKGRFTISRDNSTNTLFLQMNSLRAEDTAVYYCAR  98
M77305|IGHV3-33*03|         ADSVKGRFTISRDNSKNTLYLQMNSLRAEDTAVYYCAK  98
M77335|IGHV3-33*04|         ADSVKGRFTISRDNSKNTLYLQMNSLRAEDTAVYYCAR  98
M77334|IGHV3-33*05|         ADSVKGRFTISRDNSKNTLYLQMNSLRAEDTAVYYCAR  98
HM855436|IGHV3-33*06|       ADSVKGRFTISRDNSKNTLYLQMNSLRAEDTAVYYCAK  98
M83134|IGHV3-30*01|         ADSVKGRFTISRDNSKNTLYLQMNSLRAEDTAVYYCAR  98
L26401|IGHV3-30*02|         ADSVKGRFTISRDNSKNTLYLQMNSLRAEDTAVYYCAK  98
M99663|IGHV3-30*03|         ADSVKGRFTISRDNSKNTLYLQMNSLRAEDTAVYYCAR  98
L06615|IGHV3-30*04|         ADSVKGRFTISRDNSKNTLYLQMNSLRAEDTAVYYCAR  98
M77323|IGHV3-30*05|         ADSVKGRFTISRDNSKNTLYLQMNSLRAEGTAVYYCAR  98
L06617|IGHV3-30*06|         ADSVKGRFTISRDNSKNTLYLQMNSLRAEDTAVYYCAR  98
L06614|IGHV3-30*07|         ADSVKGRFTISRDNSKNTLYLQMNSLRAEDTAVYYCAR  98
M62737|IGHV3-30*08|         ADSVKGRFTISRDNSKNTLYLQMNSLRAEDTAVYYCAR  98
M77300|IGHV3-30*09|         ADSVKGRFAISRDNSKNTLYLQMNSLRAEDTAVYYCAR  98
M77326|IGHV3-30*10|         TDSVKGRFTISRDNSKNTLYLQMNSLRAEDTAVYYCAR  98
M77331|IGHV3-30*11|         ADSVKGRFTISRDNSKNTLYLQMNSLRAEDTAVYYCAR  98
M77338|IGHV3-30*12|         ADSVKGRFTISRDNSKNTLYLQMNSLRAEDTAVYYCAR  98
M77339|IGHV3-30*13|         ADSVKGRFTISRDNSKNRLYLQMNSLRAEDTAVYYCAR  98
M77324|IGHV3-30*14|         ADSVKGRFTISRDNSKNTLYLQMNSLRAEDTAVYYCAR  98
M77327|IGHV3-30*15|         ADSVKGRFTISRDNSKNTLYLQMSSLRAEDTAVYYCAR  98
M77328|IGHV3-30*16|         ADSVKGRFTISRDNSKNTLYLQMNSLRAEDTAVYYCAR  98
M77329|IGHV3-30*17|         ADSVKGRFTISRDNSKNTLYLQMNSLRAEDTAVYYCAR  98
X92214|IGHV3-30*18|         ADSVKGRFTISRDNSKNTLYLQMNSLRAEDTAVYYCAK  98
L06616|IGHV3-30*19|         ADSVKGRFTISRDNSKNTLYLQMNSLRAEDTAVYYCAR  98
AC244456|IGHV3-30-3*01|     ADSVKGRFTISRDNSKNTLYLQMNSLRAEDTAVYYCAR  98
M77302|IGHV3-30-3*02|       ADSVKGRFTISRDNSKNTLYLQMNSLRAEDTAVYYCAK  98
KC713945|IGHV3-30-3*03|     ADSVKGRFTISRDNSKNTLYLQMNSLRAEDTAVYYCAR  98
AC244456|IGHV3-30-5*01|     ADSVKGRFTISRDNSKNTLYLQMNSLRAEDTAVYYCAK  98
AC245243|IGHV3-30-5*02|     ADSVKGRFTISRDNSKNTLYLQMNSLRAEDTAVYYCAK  98
                            :**.****:******.* *:***.*****.*******:
```

Table S3: IgH and Igκ amino acid sequence of 1210 and 2163 antibody variants

1210 variants IgH	IGHV3-33*01 · H.31 · H.50 · H.56 · —D— · IGHJ3*02
1210_GL	QVQLVESGGGVVQPGRSLRLSCAASGFTFSSYGMHWVRQAPGKGLEWVAVIWYDGSNKYYADSVKGRFTISRDNSKNTLYLQMNSLRAEDTAVYYCARVRDSSDYYGDAFDIWGQGTMVTVSS
1210	QVQLVESGGGVVQPGRSLRLSCAASGFTFSNYGMHWVRQAPGKGLEWVAVIWYDGSKKYYADSVKGRFTISRDNSKNTLYLQMNSLRAEDTAVYYCARVRDSSDYYGDAFDIWGQGTMVTVSS
1210_H.V50_I^{mut}	QVQLVESGGGVVQPGRSLRLSCAASGFTFSNYGMHWVRQAPGKGLEWVAIIWYDGSKKYYADSVKGRFTISRDNSKNTLYLQMNSLRAEDTAVYYCARVRDSSDYYGDAFDIWGQGTMVTVSS
1210_H.N31_S^{rev}	QVQLVESGGGVVQPGRSLRLSCAASGFTFSSYGMHWVRQAPGKGLEWVAVIWYDGSKKYYADSVKGRFTISRDNSKNTLYLQMNSLRAEDTAVYYCARVRDSSDYYGDAFDIWGQGTMVTVSS
1210_H.K56_N^{rev}	QVQLVESGGGVVQPGRSLRLSCAASGFTFSNYGMHWVRQAPGKGLEWVAVIWYDGSNKYYADSVKGRFTISRDNSKNTLYLQMNSLRAEDTAVYYCARVRDSSDYYGDAFDIWGQGTMVTVSS
1210_K.N93_S^{rev}	QVQLVESGGGVVQPGRSLRLSCAASGFTFSNYGMHWVRQAPGKGLEWVAVIWYDGSKKYYADSVKGRFTISRDNSKNTLYLQMNSLRAEDTAVYYCARVRDSSDYYGDAFDIWGQGTMVTVSS
1210_H.K56_N^{rev}_K.N93_S^{rev} Named 1210_NS	QVQLVESGGGVVQPGRSLRLSCAASGFTFSNYGMHWVRQAPGKGLEWVAVIWYDGSNKYYADSVKGRFTISRDNSKNTLYLQMNSLRAEDTAVYYCARVRDSSDYYGDAFDIWGQGTMVTVSS
1210_H.D104Y_N^{mut}_K.N92_Y^{mut} Named 1210_YY	QVQLVESGGGVVQPGRSLRLSCAASGFTFSNYGMHWVRQAPGKGLEWVAVIWYDGSKKYYADSVKGRFTISRDNSKNTLYLQMNSLRAEDTAVYYCARVRDSSYYYGDAFDIWGQGTMVTVSS

1210 variants Igκ	IGKV1-5*03 · K.93 · IGKJ1*01
1210_GL	DIQMTQSPSTLSASVGDRVTITCRASQSISSWLAWYQQKPGKAPKLLIYKASSLESGVPSRFSGSGSGTEFTLTISSLQPDDFATYYCQQYNSYWTFGQGTKVEIK
1210	DIQMTQSPSTLSASVGDRVTITCRASQSISSWLAWYQQKPGKAPKLLIYKASNLESGVPLRFSGSGSGTEFTLTISSLQPDDFATYYCQQYNNYWTFGQGTKVEIK
1210_H.V50_I^{mut}	DIQMTQSPSTLSASVGDRVTITCRASQSISSWLAWYQQKPGKAPKLLIYKASNLESGVPLRFSGSGSGTEFTLTISSLQPDDFATYYCQQYNNYWTFGQGTKVEIK
1210_H.N31_S^{rev}	DIQMTQSPSTLSASVGDRVTITCRASQSISSWLAWYQQKPGKAPKLLIYKASNLESGVPLRFSGSGSGTEFTLTISSLQPDDFATYYCQQYNNYWTFGQGTKVEIK
1210_H.K56_N^{rev}	DIQMTQSPSTLSASVGDRVTITCRASQSISSWLAWYQQKPGKAPKLLIYKASNLESGVPLRFSGSGSGTEFTLTISSLQPDDFATYYCQQYNNYWTFGQGTKVEIK
1210_K.N93_S^{rev}	DIQMTQSPSTLSASVGDRVTITCRASQSISSWLAWYQQKPGKAPKLLIYKASNLESGVPLRFSGSGSGTEFTLTISSLQPDDFATYYCQQYNSYWTFGQGTKVEIK
1210_H.K56_N^{rev}_K.N93_S^{rev} Named 1210_NS	DIQMTQSPSTLSASVGDRVTITCRASQSISSWLAWYQQKPGKAPKLLIYKASNLESGVPLRFSGSGSGTEFTLTISSLQPDDFATYYCQQYNSYWTFGQGTKVEIK
1210_H.D104Y_N^{mut}_K.N92_Y^{mut} Named 1210_YY	DIQMTQSPSTLSASVGDRVTITCRASQSISSWLAWYQQKPGKAPKLLIYKASNLESGVPLRFSGSGSGTEFTLTISSLQPDDFATYYCQQYYNYWTFGQGTKVEIK

2163 variants IgH	IGHV3-33*01 · H.31 · H.50 · H.56 · —D— · IGHJ4*02
2163	QVQLVESGGGVVQPGRSLRLSCAASGFTFSSYGMHWVRQAPGKGLEWVAVIWYDGSNKYYADSVKGRFTISRDNSKNTLYLQMNSLRAEDTAVYYCARVQTTTGGGSCCPFDYWGQGTLVTVSS
2163_H.V50_I^{mut}	QVQLVESGGGVVQPGRSLRLSCAASGFTFSSYGMHWVRQAPGKGLEWVAIIWYDGSNKYYADSVKGRFTISRDNSKNTLYLQMNSLRAEDTAVYYCARVQTTTGGGSCCPFDYWGQGTLVTVSS
2163_H.S31_N^{mut}	QVQLVESGGGVVQPGRSLRLSCAASGFTFSNYGMHWVRQAPGKGLEWVAVIWYDGSNKYYADSVKGRFTISRDNSKNTLYLQMNSLRAEDTAVYYCARVQTTTGGGSCCPFDYWGQGTLVTVSS
2163_H.N56_K^{mut}	QVQLVESGGGVVQPGRSLRLSCAASGFTFSSYGMHWVRQAPGKGLEWVAVIWYDGSKKYYADSVKGRFTISRDNSKNTLYLQMNSLRAEDTAVYYCARVQTTTGGGSCCPFDYWGQGTLVTVSS
2163_H.N56_K^{mut}_K.S93_N^{mut} Named 2163_KN	QVQLVESGGGVVQPGRSLRLSCAASGFTFSSYGMHWVRQAPGKGLEWVAVIWYDGSKKYYADSVKGRFTISRDNSKNTLYLQMNSLRAEDTAVYYCARVQTTTGGGSCCPFDYWGQGTLVTVSS

2163 variants Igκ	IGKV1-5*03 · K.93 · IGKJ1*01
2163	DIQMTQSPSSLSASVGDRVTITCRASQSISSWLAWYQQKPGKAPKLLIYKASSLESGVPSRFSGSGSGTEFTLTISSLQPDDFATYYCQQYNSYWTFGQGTKVEIK
2163_H.V50_I^{mut}	DIQMTQSPSSLSASVGDRVTITCRASQSISSWLAWYQQKPGKAPKLLIYKASSLESGVPSRFSGSGSGTEFTLTISSLQPDDFATYYCQQYNSYWTFGQGTKVEIK
2163_H.S31_N^{mut}	DIQMTQSPSSLSASVGDRVTITCRASQSISSWLAWYQQKPGKAPKLLIYKASSLESGVPSRFSGSGSGTEFTLTISSLQPDDFATYYCQQYNSYWTFGQGTKVEIK
2163_H.N56_K^{mut}	DIQMTQSPSSLSASVGDRVTITCRASQSISSWLAWYQQKPGKAPKLLIYKASSLESGVPSRFSGSGSGTEFTLTISSLQPDDFATYYCQQYNSYWTFGQGTKVEIK
2163_H.N56_K^{mut}_K.S93_N^{mut} Named 2163_KN	DIQMTQSPSSLSASVGDRVTITCRASQSISSWLAWYQQKPGKAPKLLIYKASSLESGVPSRFSGSGSGTEFTLTISSLQPDDFATYYCQQYNNYWTFGQGTKVEIK

grey: CDRs | orange: mutations at positions H.31, H.50, H.56 and K.93 | blue: mutations at other positions | pink: mutations that restrict homotypic interaction by steric hindrance

Table S4: Data collection and refinement statistics

	1210-NANP$_5$	2140-1210-NANP$_3$	1450-NANP$_5$
**Wavelength (Å)**	0.97949	1.03327	0.97949
**Space group**	C2	$P4_32_12$	$C222_1$
**Cell dimensions**			
a,b,c **(Å)**	206.0, 150.9, 134.7	83.1, 83.1, 157.2	51.6, 135.1, 344.1
**α, β, γ (°)**	90, 94.8, 90	90, 90, 90	90, 90, 90
**Resolution (Å)**[a]	40-3.2 (3.3-3.2)	40-1.85 (1.95-1.85)	40-3.4 (3.6-3.4)
**No. molecules in ASU**	2	1	1
**No. unique observations**	67,565 (5,890)	47,923 (6,923)	15,950 (2,452)
**Multiplicity**	3.8 (3.8)	12.6 (12.2)	3.9 (4.0)
**R_{merge} (%)**[b]	14.7 (63.3)	6.8 (75.5)	31.1 (72.7)
**R_{pim} (%)**[c]	8.8 (37.5)	2.0 (22.3)	16.5 (38.6)
**<I/σ I>**	8.9 (1.6)	21.8 (1.7)	4.5 (1.3)
**$CC_{½}$**	99.0 (57.0)	99.9 (76.6)	95.7 (49.6)
**Completeness (%)**	99.8 (100)	99.7 (98.5)	92.1 (92.9)
**Refinement Statistics**			
**Reflections (work)**	65,564	45,843	15,107
**Reflections (test)**	2,001	2,000	796
**Non-hydrogen atoms**	13,610	3,655	6,524
**Macromolecule**	13,369	3,361	6,524
**Water**	0	269	0
**Heteroatom**	241	25	0
**R_{work}[d] /R_{free}[e]**	20.3 / 22.8	17.9 / 20.9	25.4 / 29.9
**Rms deviations from ideality**			
**Bond lengths (Å)**	0.004	0.014	0.004
**Bond angle (°)**	0.95	1.48	1.06
**Ramachandran plot**			
**Favoured regions (%)**	96.3	96.8	95.3
**Allowed regions (%)**	3.7	3.2	4.7
**B-factors ($Å^2$)**			
**Wilson B-value**	68	35	57
**Average B-factors**	83	55	89
**Average macromolecule**	83	55	89
**Average heteroatom**	92	108	-
**Average water molecule**	-	51	-

[a] Values in parentheses refer to the highest resolution bin.

[b] $R_{merge} = \Sigma_{hkl} \Sigma_i \mid I_{hkl,i} - \langle I_{hkl} \rangle \mid / \Sigma_{hkl} \langle I_{hkl} \rangle$

[c] $R_{pim} = \Sigma_{hkl} [1/(N-1)]^{1/2} \Sigma_i \mid I_{hkl,i} - \langle I_{hkl} \rangle \mid / \Sigma_{hkl} \langle I_{hkl} \rangle$

[d] $R_{work} = (\Sigma \mid |F_o| - |F_c| \mid) / (\Sigma \mid |F_o| \mid)$ for all data except as indicated in footnote e.

[e] 5% of data were used for the R_{free} calculation

Table S5: Table of contacts between $NANP_5$ and 1210 Fabs.

$NANP_5$ (BSA $Å^2$)	Interaction	1210-A	1210-B
**Ala2 (A-9 B-30)**			
Ala	VDW	H-Tyr58	H-Tyr100A
Ala^{N}	HB		H-$Tyr100A^{OH}$
**Asn3 (A-43 B-10)**			
Asn	VDW	H-Tyr58, H-Trp52, H-Tyr100A, H-Tyr100B	H-Tyr100A
**Pro4 (A-123 B-0)**			
Pro	VDW	K-Tyr94, K-Trp96, H-Trp52, H-Tyr58	
**Asn5 (A-123 B-0)**			
Asn	VDW	K-Tyr91, K-Asn92, K-Asn93, K-Tyr94, K-Trp96, H-Tyr100A, H-Tyr100B, H-Gly100C	
$Asn^{O\delta1}$	HB	K-$Tyr94^{N}$	
$Asn^{N\delta2}$	HB	K-$Asn92^{O}$	
Asn^{O}	HB	H-$Gly100C^{N}$	
**Ala6 (A-28 B-16)**			
Ala	VDW	H-Trp52, H-Tyr100A, H-Tyr100B, H-Gly100C	H-Tyr100A
**Asn7 (A-72 B-0)**			
Asn	VDW	H-Trp52, H-Val95, H-Ser98, H-Ser99, H-Asp100, H-Tyr100A, H-Tyr100B, H-Gly100C	
Asn^{N}	HB	H-$Tyr100A^{O}$	
$Asn^{N\delta2}$	HB	H-$Ser98^{O}$, H-$Asp100^{O}$	
Asn^{O}	HB	H-$Trp52^{N\varepsilon1}$	
**Pro8 (A-124 B-0)**			
Pro	VDW	H-Tyr32, H-Gly33, H-His35, H-Val50, H-Ile51, H-Trp52, H-Tyr52A, H-Val95, H-Gly100C	
Pro^{O}	HB	H-$Tyr52A^{N}$	
**Asn9 (A-108 B-0)**			
Asn	VDW	H-Asn31, H-Tyr32, H-Gly33, H-Tyr52A, H-Val95, H-Arg96, H-Asp97, H-Ser98	
$Asn^{O\delta1}$	HB	H-$Gly33^{N}$	
$Asn^{N\delta2}$	HB	H-$Val95^{O}$, H-$Arg96^{O}$,	

		H-Asp97$^{O\delta1}$	
**Ala10 (A-73 B-12)**			
Ala	VDW	H-Asn31, H-Tyr32, H-Tyr52A, H-Asp97, H-Ser98	H-Tyr58
Ala^{N}	HB	H-Asn31^{O}	
**Asn11 (A-26 B-27)**			
Asn	VDW	H-Ser98, H-Ser99, H-Asp100, H-Tyr100A, H-Tyr100B	H-Trp52, H-Tyr58, H-Tyr100B
Asn$^{N\delta2}$	HB	H-Ser99^{O}, H-Asp100^{O}	
**Pro12 (A-7 B-115)**			
Pro	VDW	H-Ser99	K-Tyr94, K-Trp96, H-Trp52, H-Tyr58
**Asn13 (A-17 B-121)**			
Asn	VDW	H-Ser99	K-Tyr91, K-Asn92, K-Asn93, K-Tyr94, K-Trp96, H-Tyr100B, H-Gly100C
Asn$^{O\delta1}$	HB		K-Tyr94^{N}
Asn$^{N\delta2}$	HB		K-Tyr94^{O}
Asn^{O}	HB		H-Gly100C^{N}
**Ala14 (A-0 B-27)**			
Ala	VDW		H-Trp52, H-Tyr100A, H-Tyr100B, H-Gly100C
**Asn15 (A-0 B-71)**			
Asn	VDW		H-Trp52, H-Val95, H-Arg96, H-Ser98, H-Asp100, H-Tyr100A, H-Tyr100B, H-Gly100C
Asn^{N}	HB		H-Tyr100A^{O}
Asn$^{N\delta2}$	HB		H-Ser98^{O}, H-Asp100^{O}
Asn^{O}	HB		H-Trp52$^{N\varepsilon1}$
**Pro16 (A-0 B-125)**			
Pro	VDW		H-Asn31, H-Tyr32, H-Gly33, H-Val50, H-Ile51, H-Trp52, H-Tyr52A, H-Val95, H-Gly100C
Pro^{O}	HB		H-Tyr52A^{N}
**Asn17 (A-0 B-107)**			
Asn	VDW		H-Asn31, H-Tyr32, H-Gly33, H-Tyr52A, H-Val95, H-Arg96, H-Asp97, H-Ser98
Asn$^{O\delta1}$	HB		H-Gly33^{N}

Asn$^{N\delta 2}$	HB	H-Val95^{O}, H-Arg96^{O}, H-Asp97$^{O\delta 1}$
**Ala18 (A-0 B-68)**		
Ala	VDW	H-Ser30, H-Asn31, H-Tyr32, H-Tyr52A
Ala^{N}	HB	H-Asn31^{O}
**Asn19 (A-0 B-51)**		
Asn	VDW	H-Ser98, H-Ser99, H-Asp100, H-Tyr100A
Asn$^{N\delta 2}$	HB	H-Ser99^{O}

HB: hydrogen bond (3.89 Å cut-off)
VDW: van der Waals (5.0 Å cut-off)

Table S6: Table of contacts between 1210 Fab-A and 1210 Fab-B.

1210-Fab (A) (BSA Å^2)	Interaction	1210-Fab (B)
**H-Tyr52A (32)**		
H-Tyr	VDW	H-Lys56
H-Tyr^{OH}	HB	H-Lys56$^{N\zeta}$
**H-Lys56 (30)**		
H-Lys	VDW	H-Tyr100A
**H-Ser99 (54)**		
H-Ser	VDW	K-Asn92, K-Asn93, H-Tyr100B
H-Ser$^{O\gamma}$	HB	K-Asn92^{O}, K-Asn93$^{O\delta1}$
H-Ser^{O}	HB	H-Tyr100B^{OH}
**H-Asp100 (45)**		
H-Asp	VDW	K-Ser30, K-Trp32, K-Asn92, H-Tyr100B
H-Asp$^{O\delta2}$	HB	K-Ser30$^{O\gamma}$, K-Asn92$^{N\delta2}$
**H-Tyr100A (104)**		
H-Tyr	VDW	K-Trp32, H-100Asp, H-Tyr100A, H-Tyr100B

HB: hydrogen bond (3.89 Å cut-off)
VDW: van der Waals (5.0 Å cut-off)

Table S7: Table of contacts between $NANP_3$ and the chimeric H.2140 / K.1210 Fab.

$NANP_3$ (BSA $Å^2$)	Interaction	H.2140 / K.1210 Fab
**Ala2 (37)**		
Ala	VDW	H-Lys56, H-Tyr58
**Asn3 (12)**		
Asn	VDW	K-Asn92, K-Asn93, H-Trp52, H-Tyr58
$Asn^{O\delta1}$	WMHB	$K\text{-}Asn92^{O}$, $K\text{-}Asn93^{O}$
$Asn^{N\delta2}$	WMHB	$H\text{-}Ser100C^{O\gamma}$
Asn^{O}	WMHB	$H\text{-}Tyr58^{OH}$
**Pro4 (117)**		
Pro	VDW	K-Asn93, K-Tyr94, K-Trp96, H-Ile50, H-Trp52, H-Tyr58
**Asn5 (132)**		
Asn	VDW	K-Tyr91, K-Asn92, K-Asn93, K-Tyr94, K-Trp96, H-Asp100B, H-Ser100C, H-Ser100D
$Asn^{O\delta1}$	HB	$K\text{-}Tyr94^{N}$
$Asn^{N\delta2}$	HB	$K\text{-}Tyr91^{O}$, $L\text{-}Tyr94^{O}$, $H\text{-}Ser100D^{O}$
Asn^{O}	HB	$H\text{-}Ser100D^{N}$
**Ala6 (14)**		
Ala	VDW	H-Trp52, H-Ser100D
**Asn7 (35)**		
Asn	VDW	H-Trp52, H-Glu97, H-Asp100B, H-Ser100D
$Asn^{N\delta2}$	WMHB	$H\text{-}Glu97^{O}$, $H\text{-}Asp100B^{O}$
Asn^{O}	HB	$H\text{-}Trp52^{N\varepsilon1}$
**Pro8 (121)**		
Pro	VDW	H-Tyr32, H-Gly33, H-Ile50, H-Ile51, H-Trp52, H-Tyr52A, H-Val95, H-Ser100D
Pro^{O}	HB	$H\text{-}Gly33^{N}$, $H\text{-}Tyr52A^{N}$
**Asn9 (85)**		
Asn	VDW	H-Ser31, H-Tyr32, H-Gly33, H-Tyr52A, H-Val95, H-Gly96, H-Glu97, H-Ser100D
$Asn^{O\delta1}$	HB	$H\text{-}Gly33^{N}$
$Asn^{N\delta2}$	WMHB	$H\text{-}Glu97^{O}$, $H\text{-}Ser100D^{O\gamma}$
**Ala10(62)**		
Ala	VDW	H-Ser30, H-Ser31, H-Tyr32, H-Tyr52A
Ala^{N}	HB	$H\text{-}Ser31^{O}$

HB: hydrogen bond (3.89 Å cut-off)
WMHB: water-mediated hydrogen bond (3.89 Å cut-off)
VDW: van der Waals (5.0 Å cut-off)

Table S8: Table of contacts between $NANP_5$ and 1450 Fabs

$NANP_5$ (BSA $Å^2$)	Interaction	1450-A	1450-B
**Asn1 (A-64 B-0)**			
Asn	VDW	K-Lys50, H-Phe98	
**Ala2 (A-65 B-0)**			
Ala	VDW	K-Trp32, H-Phe98, H-Cys99, H-Ser100	
**Asn3 (A-132 B-0)**			
Asn	VDW	K-Tyr91, H-Glu95, H-Gly96, H-Gly97, H-Phe98, H-Cys99, H-Ser100, H-Cys100D, H-Tyr100E, H-Tyr100F, H-Tyr100G	
Asn^{N}	HB	$H\text{-}Phe98^{O}$	
$Asn^{N\delta2}$	HB	$H\text{-}Glu95^{N\varepsilon2}$, $H\text{-}Gly96^{O}$, $H\text{-}Phe98^{O}$	
**Pro4 (A-122 B-0)**			
Pro	VDW	K-Trp32, H-Tyr91, H-Gly92, H-Cys100D, H-Tyr100E	
Pro^{O}	HB	$H\text{-}Tyr100E^{N}$	
**Asn5 (A-45 B-18)**			
Asn	VDW	H-Ser100A, H-Cys100D, H-Thr100C, H-Tyr100E	H-Ser100
$Asn^{O\delta1}$	HB	$H\text{-}Ser100A^{O\gamma}$	
$Asn^{N\delta2}$	HB		$H\text{-}Ser100^{O\gamma}$
**Ala6 (A-93 B-0)**			
Ala	VDW	H-Tyr58, H-Thr100C, H-Cys100D, H-Tyr100E	
Ala^{N}	HB	$H\text{-}Thr100C^{O}$	
**Asn7 (A-18 B-17)**			
Asn	VDW	H-Thr100C	H-Phe98, H-Ser100
**Pro8 (A-3 B-30)**			
Pro	VDW		K-Lys50
Pro^{O}	HB		$K\text{-}Lys50^{N\varepsilon1}$
**Asn9 (A-0 B-28)**			
Asn	VDW		K-Lys50, H-Phe98
**Ala10 (A-0 B-46)**			
Ala	VDW		H-Trp32, H-Phe98, H-Ser100
**Asn11 (A-0 B-126)**			
Asn	VDW		H-Trp32, K-Tyr91, H-Glu95, H-Gly96, H-Gly97, H-Phe98,

			H-Cys99, H-Cys100D, H-Tyr100E, H-Tyr100F, H-Tyr100G
Asn^{N}	HB		$H\text{-}Phe98^{O}$
$Asn^{N\delta2}$	HB		$H\text{-}Glu95^{N\varepsilon2}$, $H\text{-}Gly96^{O}$, $H\text{-}Phe98^{O}$
**Pro12 (A-0 B-127)**			
Pro	VDW		K-Trp32, K-Tyr91, H-Cys100D, H-Tyr100E
Pro^{O}	HB		$H\text{-}Tyr100E^{N}$
**Asn13 (A-31 B-50)**			
Asn	VDW	H-Ser100, H-Ser100A	H-Cys99, H-Ser100, H-Ser100A, H-Thr100C, H-Cys100D, H-Tyr100E
$Asn^{N\delta2}$	HB	$H\text{-}Ser100^{O\gamma}$	
$Asn^{O\delta1}$	HB		$H\text{-}Ser100A^{N}$, $H\text{-}Ser100A^{O\gamma}$
**Ala14 (A-0 B-84)**			
Ala	VDW		H-Tyr58, H-Thr100C, H-Cys100D, H-Tyr100E
Ala^{N}	HB		$H\text{-}Thr100C^{O}$
**Asn15 (A-0 B-20)**			
Asn	VDW		H-Tyr58, H-Tyr100E
Asn^{O}	HB		$H\text{-}Tyr58^{OH}$
**Pro16 (A-0 B-31)**			
Pro	VDW		H-Asp56, H-Tyr58, H-Thr100C
**Asn17 (A-0 B-79)**			
Asn	VDW		H-Gly55, H-Asp56, H-Thr57, H-Tyr58
Asn^{N}	HB		$H\text{-}Asp56^{O\delta2}$
$Asn^{O\delta1}$	HB		$H\text{-}Thr57^{N}$
$Asn^{N\delta2}$	HB		$H\text{-}Thr57^{O\gamma1}$

HB: hydrogen bond (3.89 Å cut-off)
VDW: van der Waals (5.0 Å cut-off)

Table S9: Table of contacts between 1450 Fab-A and 1450 Fab-B.

1450-Fab (A) (BSA $Å^2$)	Interaction	1450-Fab (B)
**K-Asn30 (33)**		
Asn	VDW	K-Asn30
$Asn^{N\delta2}$	HB	$K\text{-}Asn30^{\mathbf{O\delta1}}$
**H-Ser100 (25)**		
Ser	VDW	H-Ser100, H-Ser100A
**H-Ser100A (40)**		
Ser	VDW	H-Ser100, H-Ser100A
**H-Ala100B (7)**		
Ala	VDW	H-Ser100

HB: hydrogen bond (3.89 Å cut-off)
VDW: van der Waals (5.0 Å cut-off)

Table S10: BSA and contact summary for crystal structures.

Source Molecule	Target Molecule	H-bonds			BSA (Å^2)		
		H-Chain	K-Chain	Total	H-Chain	K-Chain	Total
**1210 (A)**	**$NANP_5$**	13	2	15	533	140	673
**1210 (B)**	**$NANP_5$**	13	2	15	585	122	707
**1210 (A)**	**1210 (B)**	6	0	6	259	0	259
**H.2140/K.1210**	**$NANP_3$**	7	3	10	439	130	569
**1450 (A)**	**$NANP_5$**	8	0	8	369	149	518
**1450 (B)**	**$NANP_5$**	13	1	14	460	141	601
**1450 (A)**	**1450 (B)**	0	1	1	75	33	108

References and Notes

1. F. Zavala, A. H. Cochrane, E. H. Nardin, R. S. Nussenzweig, V. Nussenzweig, Circumsporozoite proteins of malaria parasites contain a single immunodominant region with two or more identical epitopes. *J. Exp. Med.* **157**, 1947–1957 (1983). doi:10.1084/jem.157.6.1947 Medline

2. J. B. Dame, J. L. Williams, T. F. McCutchan, J. L. Weber, R. A. Wirtz, W. T. Hockmeyer, W. L. Maloy, J. D. Haynes, I. Schneider, D. Roberts, G. S. Sanders, E. P. Reddy, C. L. Diggs, L. H. Miller, Structure of the gene encoding the immunodominant surface antigen on the sporozoite of the human malaria parasite *Plasmodium falciparum*. *Science* **225**, 593–599 (1984). doi:10.1126/science.6204383 Medline

3. V. Enea, J. Ellis, F. Zavala, D. E. Arnot, A. Asavanich, A. Masuda, I. Quakyi, R. S. Nussenzweig, DNA cloning of *Plasmodium falciparum* circumsporozoite gene: Amino acid sequence of repetitive epitope. *Science* **225**, 628–630 (1984). doi:10.1126/science.6204384 Medline

4. P. Potocnjak, N. Yoshida, R. S. Nussenzweig, V. Nussenzweig, Monovalent fragments (Fab) of monoclonal antibodies to a sporozoite surface antigen (Pb44) protect mice against malarial infection. *J. Exp. Med.* **151**, 1504–1513 (1980). doi:10.1084/jem.151.6.1504 Medline

5. N. Yoshida, R. S. Nussenzweig, P. Potocnjak, V. Nussenzweig, M. Aikawa, Hybridoma produces protective antibodies directed against the sporozoite stage of malaria parasite. *Science* **207**, 71–73 (1980). doi:10.1126/science.6985745 Medline

6. L. Foquet, C. C. Hermsen, G.-J. van Gemert, E. Van Braeckel, K. E. Weening, R. Sauerwein, P. Meuleman, G. Leroux-Roels, Vaccine-induced monoclonal antibodies targeting circumsporozoite protein prevent *Plasmodium falciparum* infection. *J. Clin. Invest.* **124**, 140–144 (2014). doi:10.1172/JCI70349 Medline

7. E. M. Riley, V. A. Stewart, Immune mechanisms in malaria: New insights in vaccine development. *Nat. Med.* **19**, 168–178 (2013). doi:10.1038/nm.3083 Medline

8. B. Mordmüller, G. Surat, H. Lagler, S. Chakravarty, A. S. Ishizuka, A. Lalremruata, M. Gmeiner, J. J. Campo, M. Esen, A. J. Ruben, J. Held, C. L. Calle, J. B. Mengue, T. Gebru, J. Ibáñez, M. Sulyok, E. R. James, P. F. Billingsley, K. C. Natasha, A. Manoj, T. Murshedkar, A. Gunasekera, A. G. Eappen, T. Li, R. E. Stafford, M. Li, P. L. Felgner, R. A. Seder, T. L. Richie, B. K. L. Sim, S. L. Hoffman, P. G. Kremsner, Sterile protection against human malaria by chemoattenuated PfSPZ vaccine. *Nature* **542**, 445–449 (2017). doi:10.1038/nature21060 Medline

9. R. Murugan, L. Buchauer, G. Triller, C. Kreschel, G. Costa, G. Pidelaserra Martí, K. Imkeller, C. E. Busse, S. Chakravarty, B. K. L. Sim, S. L. Hoffman, E. A. Levashina, P. G. Kremsner, B. Mordmüller, T. Höfer, H. Wardemann, Clonal selection drives protective memory B cell responses in controlled human malaria infection. *Sci. Immunol.* **3**, eaap8029 (2018). doi:10.1126/sciimmunol.aap8029 Medline

10. G. Triller, S. W. Scally, G. Costa, M. Pissarev, C. Kreschel, A. Bosch, E. Marois, B. K. Sack, R. Murugan, A. M. Salman, C. J. Janse, S. M. Khan, S. H. I. Kappe, A. A. Adegnika, B. Mordmüller, E. A. Levashina, J.-P. Julien, H. Wardemann, Natural parasite exposure induces protective human anti-malarial antibodies. *Immunity* **47**, 1197–1209.e10 (2017). doi:10.1016/j.immuni.2017.11.007 Medline

11. D. Oyen, J. L. Torres, U. Wille-Reece, C. F. Ockenhouse, D. Emerling, J. Glanville, W. Volkmuth, Y. Flores-Garcia, F. Zavala, A. B. Ward, C. R. King, I. A. Wilson,

Structural basis for antibody recognition of the NANP repeats in *Plasmodium falciparum* circumsporozoite protein. *Proc. Natl. Acad. Sci. U.S.A.* **114**, E10438–E10445 (2017). doi:10.1073/pnas.1715812114 Medline

12. A. Ghasparian, K. Moehle, A. Linden, J. A. Robinson, Crystal structure of an NPNA-repeat motif from the circumsporozoite protein of the malaria parasite *Plasmodium falciparum*. *Chem. Commun.* **14**, 174–176 (2006). doi:10.1039/B510812H Medline

13. N. K. Kisalu, A. H. Idris, C. Weidle, Y. Flores-Garcia, B. J. Flynn, B. K. Sack, S. Murphy, A. Schön, E. Freire, J. R. Francica, A. B. Miller, J. Gregory, S. March, H.-X. Liao, B. F. Haynes, K. Wiehe, A. M. Trama, K. O. Saunders, M. A. Gladden, A. Monroe, M. Bonsignori, M. Kanekiyo, A. K. Wheatley, A. B. McDermott, S. K. Farney, G.-Y. Chuang, B. Zhang, N. Kc, S. Chakravarty, P. D. Kwong, P. Sinnis, S. N. Bhatia, S. H. I. Kappe, B. K. L. Sim, S. L. Hoffman, F. Zavala, M. Pancera, R. A. Seder, A human monoclonal antibody prevents malaria infection by targeting a new site of vulnerability on the parasite. *Nat. Med.* **24**, 408–416 (2018). doi:10.1038/nm.4512 Medline

14. J. Tan, B. K. Sack, D. Oyen, I. Zenklusen, L. Piccoli, S. Barbieri, M. Foglierini, C. S. Fregni, J. Marcandalli, S. Jongo, S. Abdulla, L. Perez, G. Corradin, L. Varani, F. Sallusto, B. K. L. Sim, S. L. Hoffman, S. H. I. Kappe, C. Daubenberger, I. A. Wilson, A. Lanzavecchia, A public antibody lineage that potently inhibits malaria infection through dual binding to the circumsporozoite protein. *Nat. Med.* **24**, 401–407 (2018). doi:10.1038/nm.4513 Medline

15. The importance of H.Y52A and H.Y58 for repeat reactivity was confirmed by alanine mutations in antibodies 1210, 2140, and 2219 (fig. S2).

16. All antibodies recognized $NANP_5$ and $NANP_3$ with binding stoichiometries of ~2 and ~1, respectively, demonstrating that $NANP_5$ but not the shorter $NANP_3$ enables binding of two Fabs.

17. C. R. Fisher, H. J. Sutton, J. A. Kaczmarski, H. A. McNamara, B. Clifton, J. Mitchell, Y. Cai, J. N. Dups, N. J. D'Arcy, M. Singh, A. Chuah, T. S. Peat, C. J. Jackson, I. A. Cockburn, T-dependent B cell responses to *Plasmodium* induce antibodies that form a high-avidity multivalent complex with the circumsporozoite protein. *PLOS Pathog.* **13**, e1006469 (2017). doi:10.1371/journal.ppat.1006469 Medline

18. B. J. DeKosky, T. Kojima, A. Rodin, W. Charab, G. C. Ippolito, A. D. Ellington, G. Georgiou, In-depth determination and analysis of the human paired heavy- and light-chain antibody repertoire. *Nat. Med.* **21**, 86–91 (2015). doi:10.1038/nm.3743 Medline

19. C. T. Watson, K. M. Steinberg, J. Huddleston, R. L. Warren, M. Malig, J. Schein, A. J. Willsey, J. B. Joy, J. K. Scott, T. A. Graves, R. K. Wilson, R. A. Holt, E. E. Eichler, F. Breden, Complete haplotype sequence of the human immunoglobulin heavy-chain variable, diversity, and joining genes and characterization of allelic and copy-number variation. *Am. J. Hum. Genet.* **92**, 530–546 (2013). doi:10.1016/j.ajhg.2013.03.004 Medline

20. T. Hattori, D. Lai, I. S. Dementieva, S. P. Montaño, K. Kurosawa, Y. Zheng, L. R. Akin, K. M. Świst-Rosowska, A. T. Grzybowski, A. Koide, K. Krajewski, B. D. Strahl, N. L. Kelleher, A. J. Ruthenburg, S. Koide, Antigen clasping by two antigen-binding sites of an exceptionally specific antibody for histone methylation. *Proc. Natl. Acad. Sci. U.S.A.* **113**, 2092–2097 (2016). doi:10.1073/pnas.1522691113 Medline

21. H. M. Davies, S. D. Nofal, E. J. McLaughlin, A. R. Osborne, Repetitive sequences in malaria parasite proteins. *FEMS Microbiol. Rev.* **41**, 923–940 (2017). doi:10.1093/femsre/fux046 Medline

22. G. Yaari, J. A. Vander Heiden, M. Uduman, D. Gadala-Maria, N. Gupta, J. N. H. Stern, K. C. O'Connor, D. A. Hafler, U. Laserson, F. Vigneault, S. H. Kleinstein, Models of somatic hypermutation targeting and substitution based on synonymous mutations from high-throughput immunoglobulin sequencing data. *Front. Immunol.* **4**, 358 (2013). doi:10.3389/fimmu.2013.00358 Medline

23. N. T. Gupta, J. A. Vander Heiden, M. Uduman, D. Gadala-Maria, G. Yaari, S. H. Kleinstein, Change-O: A toolkit for analyzing large-scale B cell immunoglobulin repertoire sequencing data. *Bioinformatics* **31**, 3356–3358 (2015). doi:10.1093/bioinformatics/btv359 Medline

24. A. P. Masella, A. K. Bartram, J. M. Truszkowski, D. G. Brown, J. D. Neufeld, PANDAseq: Paired-end assembler for Illumina sequences. *BMC Bioinformatics* **13**, 31 (2012). doi:10.1186/1471-2105-13-31 Medline

25. T. Tiller, E. Meffre, S. Yurasov, M. Tsuiji, M. C. Nussenzweig, H. Wardemann, Efficient generation of monoclonal antibodies from single human B cells by single cell RT-PCR and expression vector cloning. *J. Immunol. Methods* **329**, 112–124 (2008). doi:10.1016/j.jim.2007.09.017 Medline

26. K. Tewari, B. J. Flynn, S. B. Boscardin, K. Kastenmueller, A. M. Salazar, C. A. Anderson, V. Soundarapandian, A. Ahumada, T. Keler, S. L. Hoffman, M. C. Nussenzweig, R. M. Steinman, R. A. Seder, Poly(I:C) is an effective adjuvant for antibody and multi-functional CD4+ T cell responses to *Plasmodium falciparum* circumsporozoite protein (CSP) and αDEC-CSP in non human primates. *Vaccine* **28**, 7256–7266 (2010). doi:10.1016/j.vaccine.2010.08.098 Medline

27. W. Kabsch, XDS. *Acta Crystallogr. D* **66**, 125–132 (2010). doi:10.1107/S0907444909047337 Medline

28. A. J. McCoy, R. W. Grosse-Kunstleve, P. D. Adams, M. D. Winn, L. C. Storoni, R. J. Read, *Phaser* crystallographic software. *J. Appl. Crystallogr.* **40**, 658–674 (2007). doi:10.1107/S0021889807021206 Medline

29. P. D. Adams, P. V. Afonine, G. Bunkóczi, V. B. Chen, I. W. Davis, N. Echols, J. J. Headd, L.-W. Hung, G. J. Kapral, R. W. Grosse-Kunstleve, A. J. McCoy, N. W. Moriarty, R. Oeffner, R. J. Read, D. C. Richardson, J. S. Richardson, T. C. Terwilliger, P. H. Zwart, *PHENIX*: A comprehensive Python-based system for macromolecular structure solution. *Acta Crystallogr. D* **66**, 213–221 (2010). doi:10.1107/S0907444909052925 Medline

30. P. Emsley, B. Lohkamp, W. G. Scott, K. Cowtan, Features and development of Coot. *Acta Crystallogr. D* **66**, 486–501 (2010). doi:10.1107/S0907444910007493 Medline

31. A. Morin, B. Eisenbraun, J. Key, P. C. Sanschagrin, M. A. Timony, M. Ottaviano, P. Sliz, Collaboration gets the most out of software. *Elife* **2**, e01456 (2013). doi:10.7554/eLife.01456 Medline

32. S. H. W. Scheres, A Bayesian view on cryo-EM structure determination. *J. Mol. Biol.* **415**, 406–418 (2012). doi:10.1016/j.jmb.2011.11.010 Medline

33. S. Meixlsperger, F. Köhler, T. Wossning, M. Reppel, M. Müschen, H. Jumaa, Conventional light chains inhibit the autonomous signaling capacity of the B cell receptor. *Immunity* **26**, 323–333 (2007). doi:10.1016/j.immuni.2007.01.012 Medline

34. F. Köhler, E. Hug, C. Eschbach, S. Meixlsperger, E. Hobeika, J. Kofer, H. Wardemann, H. Jumaa, Autoreactive B cell receptors mimic autonomous pre-B cell receptor signaling and induce proliferation of early B cells. *Immunity* **29**, 912–921 (2008). doi:10.1016/j.immuni.2008.10.013 Medline

35. H. Wardemann, S. Yurasov, A. Schaefer, J. W. Young, E. Meffre, M. C. Nussenzweig, Predominant autoantibody production by early human B cell precursors. *Science* **301**, 1374–1377 (2003). doi:10.1126/science.1086907 Medline
